# Investigating Task‐Free Functional Connectivity Patterns in Newborns Using Functional Near‐Infrared Spectroscopy

**DOI:** 10.1002/brb3.70180

**Published:** 2024-12-17

**Authors:** Homa Vahidi, Alexandra Kowalczyk, Kevin Stubbs, Melab Musabi, Sriya Roychaudhuri, Michaela Kent, Soume Bhattacharya, Sandrine de Ribaupierre, Keith St. Lawrence, Yalda Mohsenzadeh, Emma G. Duerden

**Affiliations:** ^1^ Department of Neuroscience, Schulich School of Medicine and Dentistry Western University London Ontario Canada; ^2^ Division of Neonatal‐Perinatal Medicine, Department of Pediatrics London Health Sciences Centre London Ontario Canada; ^3^ Brain and Mind Centre Western University London Ontario Canada; ^4^ Department of Clinical Neurological Sciences, Schulich School of Medicine and Dentistry Western University London Ontario Canada; ^5^ Department of Medical Biophysics Western University London Ontario Canada; ^6^ Department of Computer Science Western University London Ontario Canada; ^7^ Vector Institute for Artificial Intelligence Toronto Ontario Canada; ^8^ Applied Psychology, Faculty of Education Western University London Ontario Canada

**Keywords:** connectivity, development, fNIRS, newborn

## Abstract

**Background:**

Resting‐state networks (RSNs), particularly the sensorimotor network, begin to strengthe in the third trimester of pregnancy and mature extensively by term age. The integrity and structure of these networks have been repeatedly linked to neurological health outcomes in neonates, highlighting the importance of understanding the normative variations in RSNs in healthy development. Specifically, robust bilateral functional connectivity in the sensorimotor RSN has been linked to optimal neurodevelopmental outcomes in neonates.

**Aim:**

In the current study, we aimed to map the developmental trajectory of the sensorimotor RSN in awake neonates using functional near‐infrared spectroscopy (fNIRS).

**Materials & Methods:**

We acquired fNIRS resting‐state data from 41 healthy newborns (17 females, gestational age ranging from 36 + 0 to 42 + 1 weeks) within the first week after birth. We performed both single channel and hemispheric analyses to investigate the relationship between functional connectivity and both gestational and postnatal age.

**Results:**

We observed robust positive connectivity in numerous channel‐pairs across the sensorimotor network, especially in the left hemisphere. Next, we examined the relationship between functional connectivity, gestational age, and postnatal age, while controlling for sex and subject effects. We found both gestational and postnatal age to be significantly associated with changes in functional connectivity in the sensorimotor RSN. In our hemispheric analysis (*N*_interhemispheric_ = 10, *N*_left intrahemispheric_ = 15, and *N*_right intrahemispheric_ = 9), we observed a significant positive relationship between interhemispheric connectivity and postnatal age.

**Discussion and Conclusion:**

In summary, our findings demonstrate the utility of fNIRS for monitoring early developmental changes in functional networks in awake newborns.

## Graphical abstract

The study investigates the development of resting‐state networks (RSNs) in term‐born neonates using functional near‐infrared spectroscopy (fNIRS). Results show strong positive connectivity in the sensorimotor network, particularly in the left hemisphere, and reveal that both gestational and postnatal ages are positively associated with increased functional connectivity. The findings underscore the importance of monitoring RSN development in awake infants and highlight the potential of fNIRS for studying neural activity in naturalistic settings.

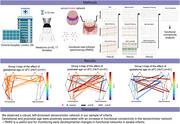

## 1 Introduction

Task‐free or resting‐state functional networks, which reflect the synchronized activity of different brain regions during rest, have been extensively studied in adult populations (Betzel et al. 2014; Power et al. 2011) and are known to play a critical role in cognitive, motor, and sensory processes (van den Heuvel and Hulshoff Pol 2010). These networks are crucial for normal brain function, as disruptions in their organization are frequently observed in various neurological and psychiatric disorders (Hull et al. 2017; Woodward and Cascio 2015). However, despite their importance, there is still a limited understanding of the relationship between task‐free functional networks and brain health, particularly early in life.

Primary functional networks, like the sensorimotor, auditory, and visual networks, have been shown to emerge in utero (Doria et al. 2010; van den Heuvel et al. 2015). In contrast, higher‐order networks such as the default mode network are thought to emerge after birth and continue to develop through the neonatal period—the first 28 days of life—and infancy (Smyser et al. 2019; Smyser and Neil 2015) before becoming well‐characterized in childhood (Cao, Huang, and He 2017). The development of the sensorimotor network is particularly important, as it is susceptible to injury during the pre‐ and perinatal periods (Eyre 2003) as well as underpinning essential functions related to sensory processing and motor execution (Liu et al. 2008).

The sensorimotor network is thought to first emerge as a unilateral network encompassing motor and sensory brain areas during the third trimester before turning into a strongly connected bilateral network at birth (Doria et al. 2010; Dall'Orso et al. 2018). During and beyond the neonatal period, sensorimotor‐network connectivity gradually decreases to a plateau as connectivity strengthens in higher‐order association areas (Collin and van den Heuvel 2013), reflecting the network's transition from an early dominant role in basic motor and sensory functions to supporting more integrated cognitive processes (Grayson and Fair 2017). The developmental shift in functional connectivity of the sensorimotor network closely mirrors synaptogenesis and synaptic pruning events in the prenatal and postnatal period (Brenner et al. 2021). More specifically, synaptogenesis of sensorimotor cortical neurons peaks around the time of birth and is followed by major synaptic pruning in the months following birth (Tau and Peterson 2010). As such, studying functional connectivity of sensorimotor network has the potential to provide us with an indirect yet valuable insight into these structural changes.

The neonatal period marks an important transition stage for sensorimotor development. Specifically, newborns are expected to encounter a multitude of novel and positive sensory and motor experiences (e.g., feeding and being held) that lay the foundation for their cognitive and behavioral development (Williams and Corbetta 2016). In contrast, newborns in critical care, particularly preterm neonates (< 37 weeks of gestation), often miss out on these positive sensory and motor experiences and instead are more likely to experience noxious sensory and motor experiences such as loud noises, painful medical interventions, and restraints (Philpott‐Robinson et al. 2017). Unsurprisingly, preterm infants are at a higher risk of poor sensory and motor outcomes later in life, a risk that is exacerbated by lower gestational age and longer duration of stay in critical care (Schanberg and Field 1987; Chorna et al. 2014; Valeri, Holsti, and Linhares 2015). Considering the aforementioned evidence, appropriate gestational age and postnatal experiences are thought to have a pivotal role in shaping normative brain development, making the characterization of the sensorimotor network in healthy newborns during this sensitive period essential to our understanding of early neurodevelopment.

Most of the current knowledge about sensorimotor resting‐state networks (RSN) and its development comes from functional magnetic resonance imaging (fMRI) studies. Yet, conducting fMRI in newborns presents unique challenges, such as newborns’ susceptibility to motion and sensitivity to loud noises. To overcome these limitations, functional near‐infrared spectroscopy (fNIRS) is increasingly used as a valuable alternative that can supplement our understanding of the developing task‐free networks, with the goal of monitoring these networks in a clinically relevant manner, particularly in neonates at risk of poor neurodevelopmental outcomes.

fNIRS allows for studies of cortical hemodynamic brain activity analogous to fMRI but in a more practical and comfortable manner, making it suitable for use in newborns, especially in clinical populations (Peng and Hou, 2021; Kebaya et al. 2023, 2024). With higher temporal resolution than fMRI, fNIRS measures changes in the concentrations of both oxygenated (HbO) and deoxygenated hemoglobin (HbR), providing a valuable proxy for neuronal activity. In addition, fNIRS can be employed for long‐term recordings (Uchitel, Vanhatalo, and Austin 2022; Uchitel et al. 2023), making it highly applicable for continuous monitoring of brain function in vulnerable neonates.

Although a few studies have investigated sensorimotor and other RSN in newborns using fNIRS (Ferradal et al. 2016; Homae et al. 2010; Kelsey et al. 2021; Taga et al. 2000), less is known about the early development of these networks in the first few days of life when the brain is especially vulnerable to injury (Eyre 2003; Nelson and Lynch 2004). Uchitel et al. (2023) used high‐density diffuse optical tomography (HD‐DOT) to examine sleep states in relation to RSNs, offering valuable insights into the intricate interactions between neural activity and sleep processes in newborns and further indicating that bedside fNIRS is highly feasible in newborns.

The sensorimotor network is expected to play a fundamental role in early sensorimotor development. More specifically, characterizing the early development of the sensorimotor network in newborns is of particular interest, as this period marks a critical time for early motor exploration and sensory experiences that shape neural connections. By investigating the associations between gestational and postnatal age—a reasonable proxy for postnatal experiences—and connectivity in the sensorimotor network, we aimed to characterize these neurodevelopmental processes specific to this sensitive period. In a heterogeneous cohort of newborns who were born in a tertiary care center, we collected fNIRS data within the first few days of life. We hypothesized that increasing gestational and postnatal age would be significantly associated with increased connectivity in the sensorimotor network in these newborns.

## 2 Methods

### 2.1 Study Setting and Participants

Participants were recruited from the Post‐Partum Care Unit (PPCU) at Victoria Hospital, London, Ontario. All infants born at term (> 37 weeks of gestation) or near‐term (> 36 weeks of gestation) who were deemed healthy by a pediatrician were eligible to participate in our study. Neonates were excluded from the study based on the following criteria: congenital malformation or syndrome, antenatal exposure to illicit drugs, postnatal infection, and suspected brain abnormalities and/or injuries. This study was approved by the Health Sciences Research Ethics Board of Western University and was conducted in accordance with the Declaration of Helsinki. All families provided written informed consent prior to data collection.

### 2.2 Demographic Data

Demographic and clinical information on maternal and newborn health were extracted from medical charts by a pediatric nurse or pediatrician. These data included gestational age, postnatal age (age since birth), sex, and head size. See Table 1 for an overview of this information.

**TABLE 1:** A table showcasing demographic and scan duration information all infants that were included in the final analysis.

Subject ID	Gestational age (weeks)	Postnatal age (h)	Sex	Head size (mm)	Total scan duration (s)	Post‐segmentation scan duration (s)	Scan remainder proportion (%)
1	40	21.5	Male		543.32	252.84	47
2	38.71	26	Female		603.59	461.24	76
3	39.14	8.5	Female		485.82	475.69	98
4	38.71	40.5	Female	360	900.86	566.43	63
5	40.14	18	Female	335	517.87	347.90	67
6	40.86	12	Male	355	723.91	423.59	59
7	38.14	9	Female	350	716.44	191.69	27
12	40	7	Female	355	390.46	311.43	80
13	40.43	21	Male	380	680.26	531.92	78
14	40.86	10	Male	355	378.27	338.85	90
15	36.43	7	Male	350	497.61	172.82	35
16	39.71	46	Female	335	577.63	159.94	28
18	40	21.5	Female	355	441.19	411.99	93
19	38.43	22	Male	340	617.55	326.57	53
24	40.14	13	Male	370	493.88	390.66	79
25	40.57	15.5	Male	350	484.93	364.71	75
27	39	24	Male	350	387.91	328.73	85
31	36.14	20	Female	345	460.95	154.83	34
33	38.86	32	Male	315	622.17	373.65	60
35	36.86	32	Female	345	435.59	231.31	53
36	41.14	20	Male	345	609.19	520.72	85
37	39	11	Male	365	569.28	214.79	38
38	38.71	22	Male	380	481.89	415.92	86
39	39.71	19.25	Female	350	616.76	454.75	74
40	38.29	19	Male	310	613.81	409.73	67
44	40.71	21	Female	340	695.50	315.65	45
45	38.14	5	Male	340	616.66	151.78	25
47	39	29	Female	350	618.92	573.60	93
48	38.86	33	Female	330	552.86	357.73	65
49	39.71	19	Male	340	722.63	213.91	30
50	39	24	Male	320	662.86	588.64	89
51	38.43	54	Male	360	604.96	381.71	63
52	39.86	23	Male	345	599.95	381.62	64
53	37.57	26	Female	340	603.59	385.16	64
54	36.43	33	Male	330	593.46	351.63	59
56	39	22.5	Male	370	597.88	177.73	30
57			Male	376	601.52	375.42	62
58	37.43	30	Male	340	620.20	445.61	72
59	39.86	21	Male	340	611.94	573.60	94
60	41.29	31	Female	345	636.72	579.40	91
61	38	48	Female	320	619.91	403.83	65

*Note*: Gestational age was calculated by converting the raw gestational ages (e.g., 38 weeks + 1 day) to a single number (38.1) rounded to the first significant digit. Empty cells represent missing data. To limit motion–related noise in our data, we used segmentation to isolate segments of data that were not contaminated by motion. Post‐segmentation scan duration represents the sum of the duration of all these segments and scan remainder proportion represents the proportion of the total segment length to the total scan time for each infant.

### 2.3 fNIRS Data Collection

Upon entering the PPCU, the healthcare team identified any families whose newborn would be eligible to participate in our study. All eligible families were first approached by their primary nurse to give verbal consent to be approached by researchers. After receiving verbal consent, a member of the research team approached every family, explained the nature of the study, presented families with a voided copy of the informed consent form, and gave them as much time as they requested to consider enrolling their newborn in the study. After receiving written informed consent, the newborns’ head circumferences were measured using a measuring tape and fit with an optode‐prepopulated, properly sized fNIRS cap (Easycap GmbH, Germany). We measured the nasion‐to‐inion distance and positioned the cap so that Cz was centered along the anterior–posterior axis of the head. To ensure left–right symmetry, we aligned Cz midway between the left and right ear canals. Since the Easycaps come in even sizes, for infants with an odd head circumference, we chose to size up to the nearest cap size to minimize discomfort. When possible, we recorded data after infants were fed and were most calm to decrease the likelihood of motion and general fussiness. Although it is difficult to accurately comment on each infant's sleep state without acquiring an electroencephalogram, all infants were awake when being fitted with the cap and likely remained awake for the short data recording duration immediately after. We did not conduct any photography or video recordings of infants during any portion of the data collection.

**FIGURE 1:**
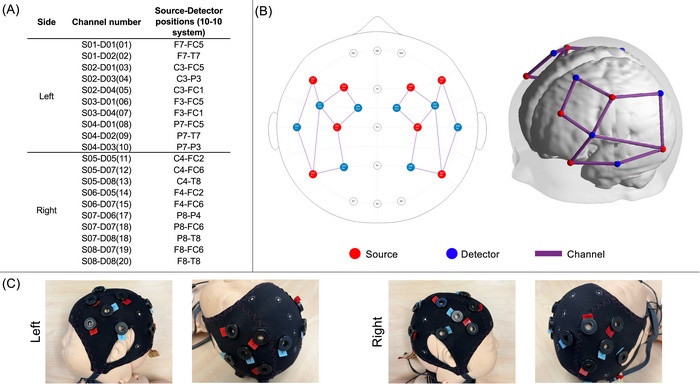
(A) 10–10 Locations of source‐detector pairs. (B) 2D and 3D views of the montage used. Sources are shown in red, detectors are shown in blue, and channels between them are shown in purple. (C) A model of a newborn wearing an fNIRS cap setup with our montage.

We recorded task‐free fNIRS signal from 61 infants at bedside using a multichannel NIRSport2 system (NIRStar Software v14.0, NIRx Medical Technologies LLC, Berlin, Germany) at a sampling rate of 10.17 Hz. Our optode setup included eight LED sources (760 and 850 nm) and eight detectors, which yielded 20 channels (10 per hemisphere) with an average source‐detector separation of 35.7 ± 9.5 mm (see Figure 1). Previous studies investigating the minimum resting‐state fNIRS imaging duration for accurate and stable mapping of brain connectivity networks in children recommended a minimum of 2.5 min recording duration (J. Wang, Dong, and Niu 2017). Therefore, when possible, data were recorded for a minimum of 10 min in each newborn to ensure stable and accurate functional connectivity calculations. Data from four participants were excluded from preprocessing and analysis due to system malfunction and suboptimal calibration at the time of data collection. The remaining 57 (93%) infants comprised of 25 female and 32 male newborns with a mean gestational age of 39.01 ± 1.21 weeks and a mean postnatal age of 23.66 ± 11.75 h.

### 2.4 fNIRS Preprocessing and Quality Assurance

All data pruning, preprocessing, and analysis were performed with MATLAB 2022b (The MathWorks Inc., Natick, MA) using AnalyzIR Toolbox (Santosa et al. 2018), Homer2 (Huppert et al. 2009), QT‐NIRS (Hernandez and Pollonini 2020), and in‐house scripts.

#### 2.4.1 Channel/Participant Screening and Exclusion

First, raw data were transformed to optical density. Second, all channels in the 57 datasets were manually screened for the presence of cardiac pulsation. Specifically, we surveyed patterns in the frequency domain to flag channels that did not have signal in the expected frequency range for cardiac pulsation in infants (Southall et al. 1980). We excluded 12 datasets where no channels had detectable cardiac pulsation. See Figure S1 for a group spatial map of all remaining channels. Third, to further account for motion artifacts present in our data, we chose to segment out any prolonged periods of motion from every dataset. To do so, for each of the remaining 45 datasets, we used QT‐NIRS (Hernandez and Pollonini 2020) to calculate scalp coupling index (SCI) and peak spectral power (PSP) in 5‐s windows with 4‐s overlaps for a cardiac range of 60–210 beats per minute, encompassing the 70–200 beats per minute cardiac range commonly reported in neonates (Southall et al. 1980). For each dataset, we empirically defined a SCI threshold of 0.1 and a PSP threshold of 0.03 to identify motion‐free segments of at least 50 s (Bulgarelli et al. 2024). Brief motion artifacts (less than 2 s) were ignored during segmentation and later corrected for using filtering methods. Although the SCI and PSP thresholds used were considerably lower than the defaults introduced in QT‐NIRS (Hernandez and Pollonini 2020), we evaluated several different values (by visually inspecting each dataset before and after segmentation) and selected the most appropriate thresholds for our sample. Datasets were excluded if they did not produce any > 50‐s motion‐free segments or if they had a total segment length < 2.5 min, the minimum recommended dataset length for fNIRS resting‐state functional connectivity analysis in infants (J. Wang, Dong, and Niu 2017). This led to the removal of 4 datasets, leaving 41 datasets for further preprocessing and analysis. Table 1 shows the demographic information for the remaining sample of 41 infants.

The aforementioned steps led to a total of 16 (28%) datasets being excluded from further analyses. The remaining sample (*n* = 41, 17 females) had a mean gestational age of 39.08 ± 1.31 weeks and a postnatal age of 22.93 ± 11.20 h. We performed a *χ* ^2^ test of independence to identify any sex differences between our dataset before and after participant exclusions. We additionally performed two two‐sample *t* tests to identify any significant differences in gestational and postnatal ages. We found no statistically significant differences between the original dataset and the post‐exclusion dataset.

#### 2.4.2 Preprocessing Pipeline

**FIGURE 2:**
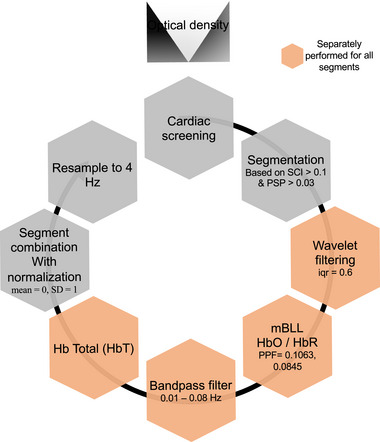
Overview of fNIRS data preprocessing steps. The black circular arrow indicates processing order starting with cardiac screening and ending with resampling to 4 Hz before sFC analysis.

For each dataset, the motion‐free segments were preprocessed independently and recombined at the end of the preprocessing pipeline. For each segment, AnalyzIR Toolbox's wavelet filtering method with a standard deviation threshold of 0.8 (equivalent to a Homer2's interquartile range of 0.6) was applied to remove short motion spikes and slow drifts (Ravicz et al. 2015; Di Lorenzo et al. 2019). Next, wavelet filtered optical density data were transformed to estimated changes in HbO and HbR concentrations using the modified Beer–Lambert law (mbLL) (Santosa et al. 2018). When applying the mbLL, age‐appropriate partial pathlength factors were used (0.1063 for 760 nm and 0.0845 for 850 nm) (Scholkmann and Wolf 2013), and channel lengths were scaled to the cap size used. Next, a bandpass filter (0.01–0.08 Hz) was applied to HbO and HbR separately to remove low‐frequency system noise and any remaining physiological noise like respiration and Mayer waves (Eggebrecht et al. 2014). The data were then converted to estimated change in total hemoglobin concentration (HbT = HbO + HbR), which has been shown to have improved functional connectivity reproducibility across participants (Novi, Rodrigues, and Mesquita 2016). Next, for each dataset, we normalized (mean = 0, SD = 1) and combined all segments. The final data were resampled to 4 Hz before calculating spontaneous functional connectivity (sFC). Figure 2 and Appendix 1 in Supporting Information show an overview of the preprocessing steps and plots of an example dataset at every stage of preprocessing, respectively. We also analyzed HbO and HbR separately. For those analyses, apart from calculating HbT, all the other steps of preprocessing and analysis remained the same.

### 2.5 Analysis Pipeline

#### 2.5.1 Spontaneous Functional Connectivity

We used AnalyzIR Toolbox's correlation method to calculate sFC. More specifically, for each dataset, we created a 20 × 20 symmetric correlation matrix by calculating Pearson's R correlation coefficients between the time series of all possible channel‐pairs. Correlation coefficients were then *z*‐transformed. We performed a single‐sample *t*‐test for each unique channel‐pair evaluating sFC > 0 across the entire sample.

#### 2.5.2 Relating sFC to Gestational Age and Postnatal Age

We used linear mixed effects (LME) modeling to evaluate the relationship between sFC and both gestational and postnatal age while accounting for variability due to biological sex and between‐subject effects. Modeling was performed independently for each channel pair and on groups of channel‐pairs located intra‐ and interhemispherically. Gestational age and postnatal age were included as fixed variables, whereas subject ID and sex were included as random and grouping variables. For single channel‐pair analyses, each channel‐pair's model produced a *t* value and *p* value for both gestational age and postnatal age, which indicates their linear relation to sFC across the sample. For hemisphere‐wide analyses, three models were evaluated, which corresponded to channel‐pairs located on the left hemisphere, channel‐pairs located on the right hemisphere, and channel‐pairs spanning both hemispheres, respectively.

#### 2.5.3 Statistical Analysis

All statistical analyses were performed using MATLAB. We explored the relationship between sFC and both gestational age and postnatal age. For individual channel‐pair analyses, we corrected our findings for multiple comparisons using the false discovery rate with a *q* value set at 0.05. We used the number of channel‐pairs (*n* = 190) to calculate the degrees of freedom. For hemisphere‐wide analyses, since we used three separate LME models, we used a Bonferroni‐corrected threshold of significance (*p* < 0.02).

## 3 Results

### 3.1 Exploring Single Channel Spatial Patterns in sFC

**FIGURE 3:**
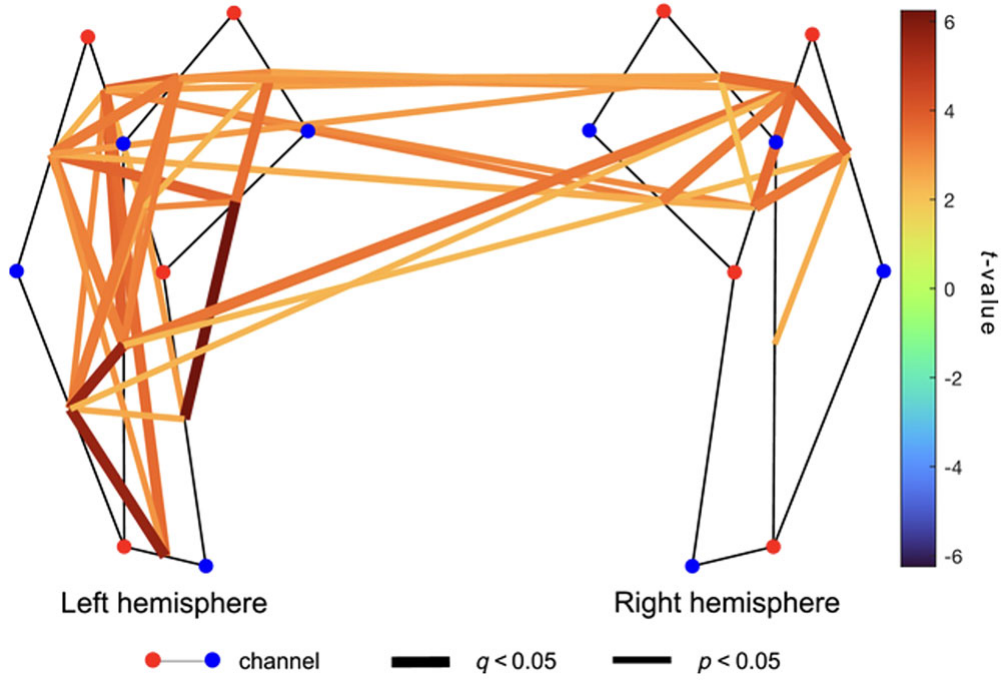
Group *t*‐map showing spontaneous functional connectivity (sFC) spatial patterns for HbT (*n* = 41). Channel‐pairs displaying a significant positive or negative sFC are depicted in red and blue lines, respectively. The false discovery rate (FDR) was used to correct for multiple comparisons. Channel‐pairs that exhibited significant connectivity after FDR correction are drawn as thick lines, whereas channel‐pairs with significant connectivity before FDR correction are denoted with thin lines. The color of the lines represents the *t*‐value calculated for that channel‐pair's connectivity. Channel‐pairs that had fewer than 10 datapoints and those that were not significant have been omitted to increase clarity.

First, we examined the presence of any existing spatial sFC patterns in our sample. We observed several significant and positively correlated intra‐ and interhemispheric channel‐pairs, especially in the left hemisphere (Figures 3 and S2). Specifically, we observed positive connectivity (*p* < 0.05) in 32 of 45 (∼71%) of all possible intrahemispheric channel‐pairs in the left hemisphere, 20 of 45 (∼44%) of all possible intrahemispheric channel‐pairs in the right hemisphere, and 27 of 100 (27%) of all possible interhemispheric channel‐pairs. The sFC patterns in HbT were comparable to those seen in HbO (see Figures S3 and S4). The sFC patterns in HbR were generally much weaker (see Figures S5 and S6).

### 3.2 Relating sFC to Gestational Age and Postnatal Age

**FIGURE 4:**
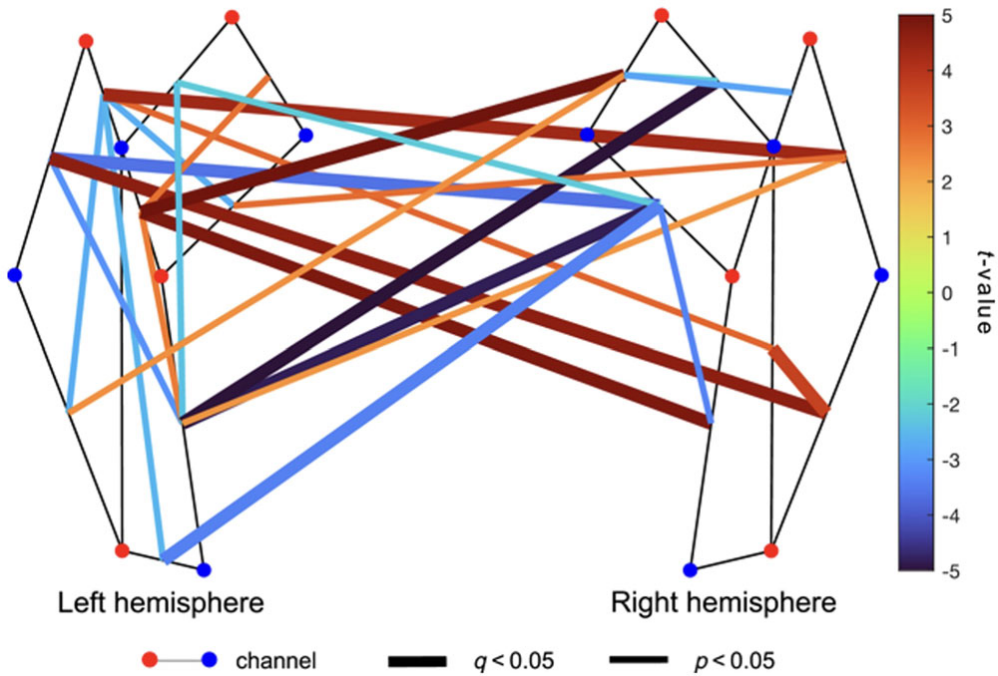
Group regression analysis demonstrating gestational age–related patterns in spontaneous functional connectivity (sFC) (*n* = 41). Channel‐pairs displaying a significant positive or negative effect of gestational age on sFC are depicted in red and blue lines, respectively. The false discovery rate (FDR) was used to correct for multiple comparisons. Channel‐pairs that exhibited significant connectivity after FDR correction are drawn as thick lines, whereas channel‐pairs with significant connectivity before FDR correction are denoted with thin lines. The color of the lines represents the *t*‐value calculated for that channel‐pair's connectivity. Channel‐pairs that had less than 10 datapoints and those that were not significant have been omitted to increase clarity.

We identified several channel‐pairs within and across the left and right hemispheres where increasing gestational age was significantly associated with both increasing and decreasing sFC (Figures 4 and S7). Specifically, increasing gestational age was significantly associated with increasing sFC in 11 of 100 interhemispheric, 1 of 45 left intrahemispheric, and 1 of 45 right intrahemispheric channel‐pairs. However, increasing gestational age was significantly associated with decreasing sFC in 8 of 100 interhemispheric, 5 of 45 left intrahemispheric, and 4 of 45 right intrahemispheric channel‐pairs.

**FIGURE 5:**
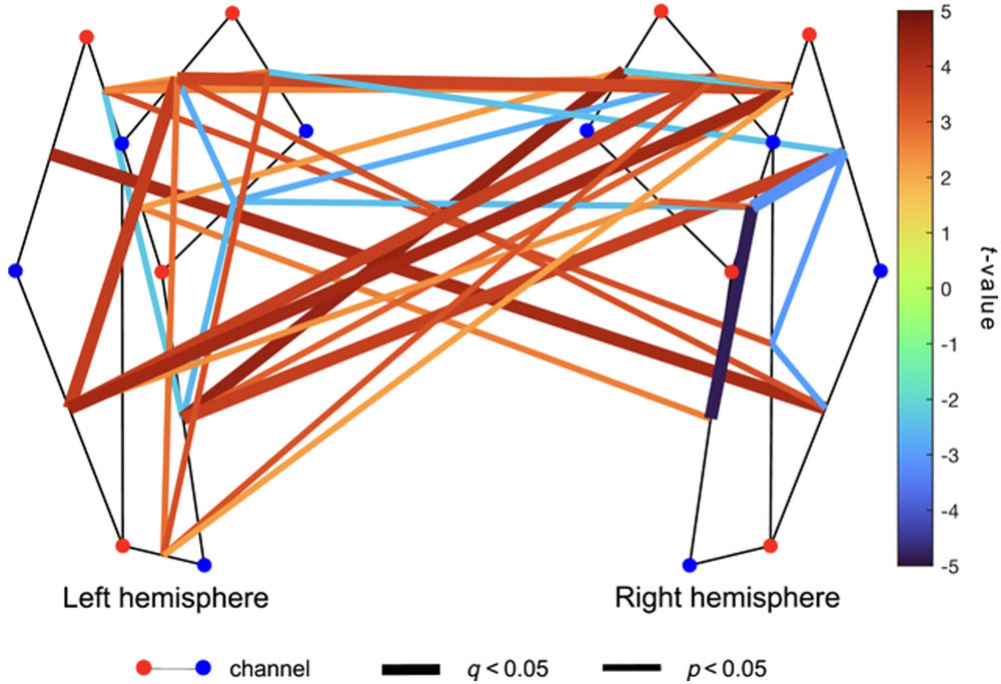
Whole montage regression analysis demonstrating postnatal age–related patterns in functional connectivity (sFC) (*n* = 41). Channel‐pairs displaying a significant positive or negative effect of postnatal age on sFC are depicted in red and blue lines, respectively. The false discovery rate (FDR) was used to correct for multiple comparisons. Channel‐pairs that exhibited significant connectivity after FDR correction are drawn as thick lines, whereas channel‐pairs with significant connectivity before FDR correction are denoted with thin lines. The color of the lines represents the *t*‐value calculated for that channel‐pair's connectivity. Channel‐pairs that had fewer than 10 datapoints and those that were not significant have been omitted to increase clarity.

We also identified several intra‐ and interhemispheric channel‐pairs where increasing postnatal age was mainly associated with increasing sFC (Figures 5 and S8). Specifically, increasing postnatal age was significantly associated with increasing sFC in 18 of 100 interhemispheric, 4 of 45 left intrahemispheric, and 5 of 45 right intrahemispheric channel‐pairs. However, increasing postnatal age was significantly associated with decreasing sFC in 4 of 100 interhemispheric, 2 of 45 left intrahemispheric, and 2 of 45 right intrahemispheric channel‐pairs.

### 3.3 Exploring Hemisphere‐Wide Changes in sFCas a Function of Gestational and Postnatal Age

**FIGURE 6:**
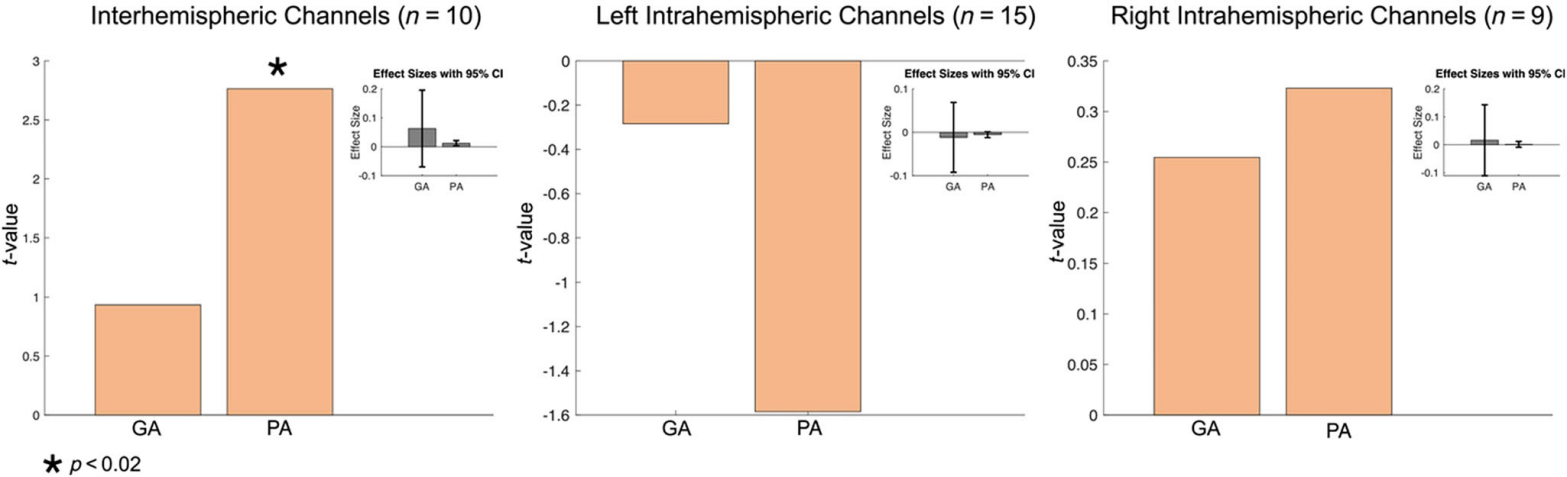
Hemisphere‐wide regression analyses demonstrating gestational age– and postnatal age–related patterns in functional connectivity. From left to right, each plot illustrates the effect of gestational age (GA) and postnatal age (PA) on interhemispheric, intrahemispheric (left), and intrahemispheric (right) functional connectivity. Only participants who had more than 50% of channels present after channel pruning were included in the analysis. The number of participants included is denoted on top of each plot. Each plot also includes a mini plot representing the effect sizes with 95% confidence intervals (CI). Significant effects are denoted by a star.

Next, we attempted to characterize the relationship between gestational and postnatal age and functional connectivity across all channel‐pairs. We divided all channel‐pairs into interhemispheric (i.e., channel‐pairs connecting the left and right hemispheres), left intrahemispheric (i.e., channel‐pairs where both channels are located in the left hemisphere), and right intrahemispheric. Then, a linear mixed‐effects model was used to evaluate the relationship between gestational and postnatal age and connectivity in each of the channel‐pair groups. We observed a significant positive association between postnatal age and functional connectivity in interhemispheric channels, *t*(10) = 2.76, *p* = 0.0059. None of the other associations were found to be statistically significant (Figure 6).

## 4 Discussion

We evaluated functional changes in sensorimotor RSN connectivity in the first few days of life in a sample of 41 healthy near‐term and term‐born newborns. We conducted task‐free fNIRS recordings in these newborns within the first few days of life at bedside.

Similar to previous fMRI and fNIRS studies in neonates (Doria et al. 2010; Turk et al. 2019; Uchitel et al. 2023), we observed strong positive interhemispheric and intrahemispheric connectivity in the sensorimotor network. Interestingly, we observed the strongest connectivity between channel‐pairs within the left hemisphere. It is widely known that human sensorimotor functions are strongly left lateralized depending on handedness preference (Janssen, Meulenbroek, and Steenbergen 2011). As more than 90% of the general population prefers using their right hand to perform most motor tasks (McManus 2019), this lateralization may be determined early on in development (Erberich et al., 2006). In fact, ultrasound monitoring of fetal arm movements has indicated a potential asymmetry in hand preference as early as the second trimester (Parma et al. 2017). In addition, compared to newborns born to left‐handed or ambidextrous families, newborns of right‐handed parents and siblings were significantly more likely to exhibit left cerebral dominance when tested for motor and sensory functions (Cioni and Pellegrinetti 1982), further pointing toward the presence of functional asymmetry in the sensorimotor network early in life. As such, the strong left‐dominant sensorimotor connectivity we observe in our sample further highlights this functional asymmetry in the developing sensorimotor network. Contrary to our findings, a previous study using frequency‐domain near‐infrared spectroscopy (FDNIRS) and diffuse correlation spectroscopy (DCS) showed a right‐hemispheric dominance in blood flow in a large sample of preterm and term‐born newborns. However, while the sample investigated in this study was mostly comprised of preterm infants, our sample primarily included term‐born infants, with only two infants being near‐term (Lin et al. 2013). As such, the asymmetry in functional connectivity of the sensorimotor network seen in our sample, which directly relates to cerebral blood flow, could be due to our sample capturing a different window in healthy development than the one captured by Lin et al. (2013). Alternatively, as previously mentioned in our methods, any channels without clear cardiac pulsation were removed from further analysis. This process led to more channels being removed from the right hemisphere compared to the left (see Figure S1). Although at least 10 or more datasets were included per channel, the overall lower number of viable datapoints on the right hemisphere could partially explain the asymmetry seen between connectivity patterns in the left and the right hemispheres.

We further examined the relationship between gestational age, postnatal age, and functional connectivity in the sensorimotor network. Contrary to our hypothesis, older gestational ages were associated with only a modest increase in bilateral connectivity in the sensorimotor network, with many interhemispheric channel‐pairs displaying a negative effect of gestational age. The positive relationship observed between gestational age and connectivity was somewhat weaker than those reported in previous fMRI studies (Doria et al. 2010). Our findings could be due to the older and narrower gestational age range of our sample compared to previous studies. More specifically, previous fMRI and fNIRS studies reporting a strong positive relationship between gestational age and sFC have included preterm neonates and thus had a much wider gestational age range, with most neonates being preterm (Doria et al. 2010; Smyser et al. 2019). Preterm development of cortical RSNs such as the sensorimotor network is reflective of synchronous maturation of cortical gray matter and white matter. Essential to this process in utero, and during the preterm period, is subplate connectivity reflected in synaptogenesis and thalamo‐cortical projections. This period is marked by rapid synaptogenesis, which is later offset by neuronal apoptosis and pruning of weaker synapses (Petanjek et al. 2008; Shatz 1996). In addition, a key factor in RSN development is myelination. Broadly, subcortical areas begin to myelinate mid‐gestation, followed by the posterior cortex and frontal cortex (Jakovcevski 2009). In the cerebral cortex, myelination begins in the central sulcus and extends toward the posterior cortex, followed by the frontotemporal locations (Levitt 2003). The structural and functional maturation of these processes are shaped by spontaneous neuronal activity (e.g., motor responses) and external stimuli such as sensory input in the extrauterine environment (Huttenlocher 2002; Petanjek et al. 2008). Since the processes occur in the span of months before birth, a narrower gestational age range will greatly impact the developmental window being observed, further explaining our findings. In turn, since we aimed to capture age‐related changes in functional connectivity in a manner that reflected normative development, our sample of healthy infants was inherently undergoing fewer maturational changes that could be evidenced with fNIRS.

In our sample, postnatal age (i.e., age since birth) was associated with a widespread increase in interhemispheric connectivity across channels covering the sensorimotor network. Findings may reflect increased neuronal activity in the postnatal environment due to exposure to novel environmental stimuli in the first few days of life. Ferradal et al. (2016), utilizing HD‐DOT, recorded task‐free oscillations in brain activity in newborns during a similar postnatal period (i.e., within the first 2 days of life). They similarly reported strong interhemispheric connectivity in middle temporal, visual, and auditory RSNs (Ferradal et al. 2016). However, limited intrahemispheric connectivity was reported for the same networks. Although our findings were primarily localized to the territory of the sensorimotor network, we found increasing interhemispheric and some evidence for left‐sided intrahemispheric connectivity (at the channel‐level analyses only) at older postnatal ages in this the sensorimotor RSN, even during periods of natural sleep/rest. Intrahemispheric connectivity may be a key factor supporting hemispheric specialization (Tzourio‐Mazoyer 2016). The first few days of life are characterized by jerky and non‐goal‐directed general movements, in addition to early motor reflexes (Lenard, von Bernuth, and Prechtl 1968), whereas the first few weeks of life are characterized by the development of coordinated motor movements (Hannan and Fogel 1987). Absence of these sensorimotor behaviors may be an indicator of adverse neurodevelopment later in life (Gajewska et al. 2013; Hadders‐Algra 2004). In turn, the use of fNIRS at the bedside may identify early biomarkers for typical sensorimotor development.

### 4.1 Study Limitations

Our study included a heterogeneous sample of day‐old newborns who were tested with a standardized fNIRS protocol. Despite the challenges faced by recruiting this vulnerable population, our results provide evidence for the emergence of robust RSNs as well as inter‐ and intrahemispheric connectivity that aligns with brain maturational stages. However, our study had several limitations that are inherent to fNIRS data collection in infants.

First, we excluded datasets and/or channels of subpar quality that led to variable number of viable channels in each dataset. The regression method we employed to assess the relationship between sFC and both gestational and postnatal age accounted for the number of channel‐pairs used. Nonetheless, it is advisable for future studies to employ larger sample sizes to corroborate and expand upon our findings.

Second, our montage was confined to motor, premotor, and sensory cortical regions. Consequently, we were unable to examine developmental changes in other RSNs during this crucial phase. Prior fMRI studies involving newborns have showcased consistent and robust developmental pathways for networks encompassing sensory and motor regions (e.g., sensorimotor, auditory, and visual networks) (Doria et al. 2010; Dall'Orso et al. 2018). However, the developmental trajectories identified for more complex networks (e.g., the Default Mode Network) are more variable and tend to be challenging to examine due to the coarse spatial resolution of fNIRS. As a result, for the scope of this study, we opted to concentrate solely on the sensorimotor network; however, future work could also include whole‐head coverage to better characterize the sensorimotor networks in relation to other RSNs to better understand early cortical connectomics.

Third, the source‐detector distances used in this study were larger and more variable than those typically used in neonates and young infants (Kelsey et al. 2021). Although these larger distances provided broader coverage of the sensorimotor areas, previous research in adults has shown that increasing source‐detector distances is negatively correlated with the signal‐to‐noise ratio (SNR) (Y. Wang and Chen 2020). In this study, we did not observe significant changes in SNR, the ratio of pruned channels, or SCI as a function of source‐detector distance. Nonetheless, future research is needed to systematically evaluate and determine optimal source‐detector distances, specifically across different brain areas and in neonates.

Fourth, newborns’ sensory and motor development were not assessed. All newborns enrolled in this study were examined by a pediatrician and were healthy in relation to their sensory and motor reflexes. Furthermore, subject‐related effects were accounted for in our regression model. Incorporating standardized neurodevelopmental assessments could expand on how sensorimotor RSN characteristics predict motor outcomes.

Finally, although we noted that all infants were awake when being fitted with the cap and likely remained awake immediately after for the duration of data collection, we did not systematically monitor sleep states (e.g., using electroencephalography and/or video/photo recordings). A number of studies suggest that sleep states (e.g., active vs. quiet sleep) may modulate functional connectivity in infants (Uchitel, Vanhatalo, and Austin 2022; Uchitel et al. 2023). As such, future studies, especially in the neonatal population, should attempt to monitor alertness to help increase the interpretability of their findings.

## 5 Conclusions

This study aimed to advance our understanding of the development of sensorimotor RSNs in healthy newborns using fNIRS. By exploring functional connectivity within the sensorimotor RSNs and how it is associated with gestational and postnatal age, this study contributes to our understanding of normative brain development during early yet important stages of life. Although our study demonstrates the utility of using fNIRS at the bedside in neonates, our findings also highlight the challenges associated with fNIRS data collection and analysis in postpartum care centers. Given the utility of fNIRS in healthy newborns and neonates impacted by critical illness, our study highlights the need for improved fNIRS methodologies tailored for this vulnerable population.

## Author Contributions

 HV, AK, SB, S de R, KSL, YM and EGD were involved in the conceptualization and design of the study. HV, AK, MM, SR, and MK were involved in data collection and curation. HV, KS, MK, KSL, YM and EGD were involved in the analysis. HV and KS prepared the figures. HV, YM and EGD wrote the draft of themanuscript. All all authors read the final version of the manuscript.

## Conflicts of Interest

The authors declare no conflicts of interest.

### Peer Review

The peer review history for this article is available at https://publons.com/publon/10.1002/brb3.70180.

## Supporting information



Appendix 1. An overview of an example dataset at every processing stage.

Figure S1. Group spatial map of channel exclusions (*n* = 41).
Figure S2. Matrix showing group spontaneous functional connectivity (sFC) for HbT (*n* = 41).
Figure S3. Matrix showing group spontaneous functional connectivity (sFC) for HbO (*n* = 41).
Figure S4. Group t‐map showing spontaneous functional connectivity (sFC) spatial patterns for HbO (*n* = 41).
Figure S5. Matrix showing group spontaneous functional connectivity (sFC) for HbR (*n* = 41).
Figure S6. Group t‐map showing spontaneous functional connectivity (sFC) spatial patterns for HbR (*n* = 41).
Figure S7. Matrix demonstrating gestational age–related patterns in spontaneous functional connectivity (sFC) (*n* = 41).
Figure S8. Matrix demonstrating postnatal age–related patterns in spontaneous functional connectivity (sFC) (*n* = 41).

## Data Availability

The data and code used in this study are available upon request.
