# Supplementary material for: Investigating Task‐Free Functional Connectivity Patterns in Newborns Using Functional Near‐Infrared Spectroscopy
Source: Brain Behav. 2024 Dec 17;14(12):e70180. doi: 10.1002/brb3.70180 (PMC11652786; doi:10.1002/brb3.70180)

Appendix 1. An overview of an example dataset at every processing stage. This figure includes a representative dataset from one participant. Each plot in the “Time Courses” panel represents the time series at that stage of preprocessing. Note that before HbO and HbR calculations, each channel’s data is comprised of 2 wavelengths (760 and 850 nm) that are plotted separately. After HbO and HbR concentrations are calculated, each channel’s data is comprised of HbO and HbR concentrations that are also plotted separately. Once HbO and HbR signals are combined to calculate HbT, each channel is reduced to a single trace. Under the “Correlations Plots” panel, we have calculated a correlation matrix at every stage (separately for each segment when more than one segment is present) to explore how the relationship between channels changes at every stage of preprocessing.

Raw

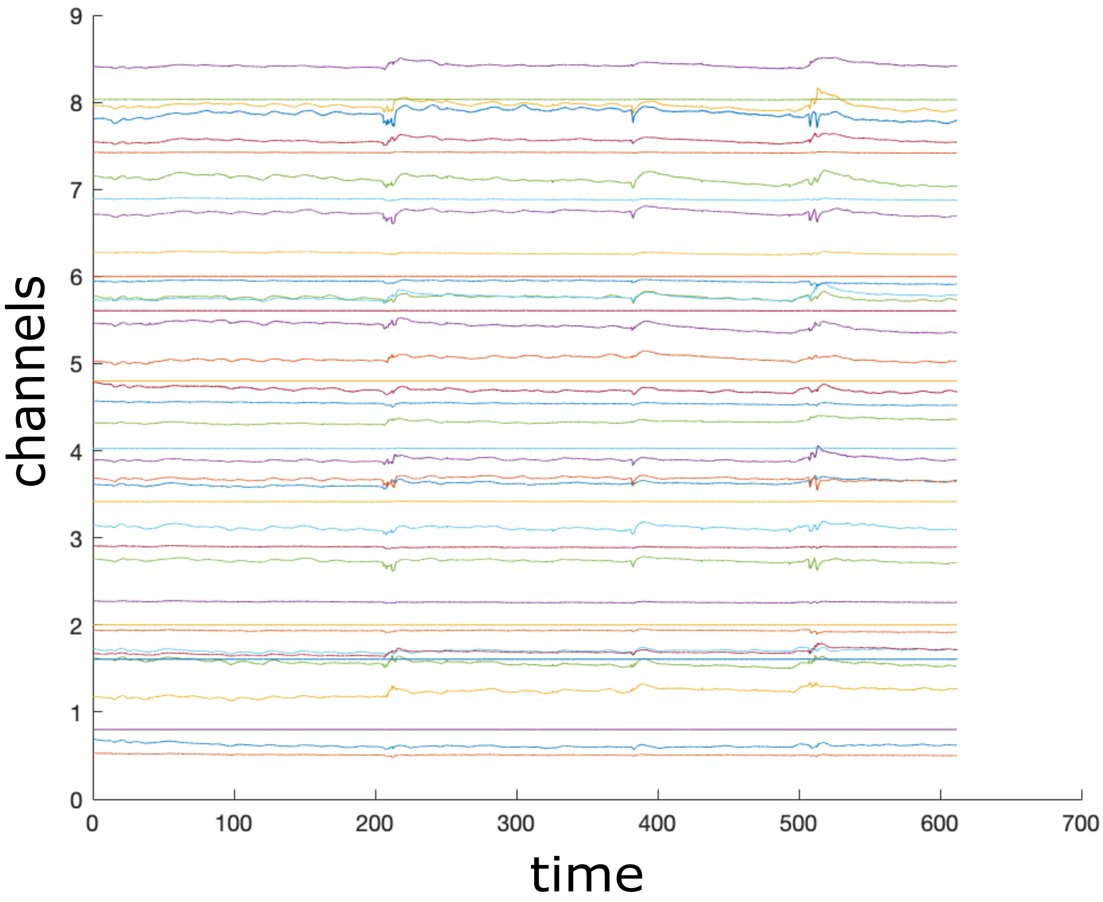

Correlation Plots

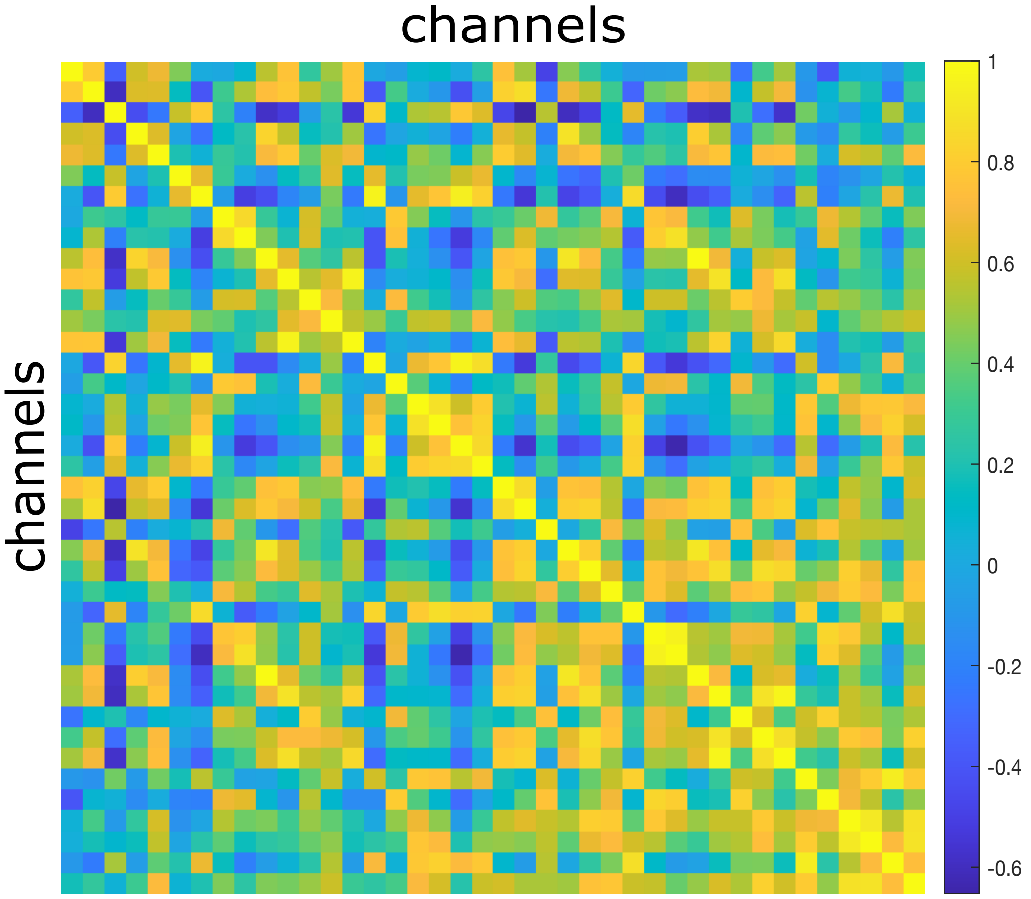

Optical Density

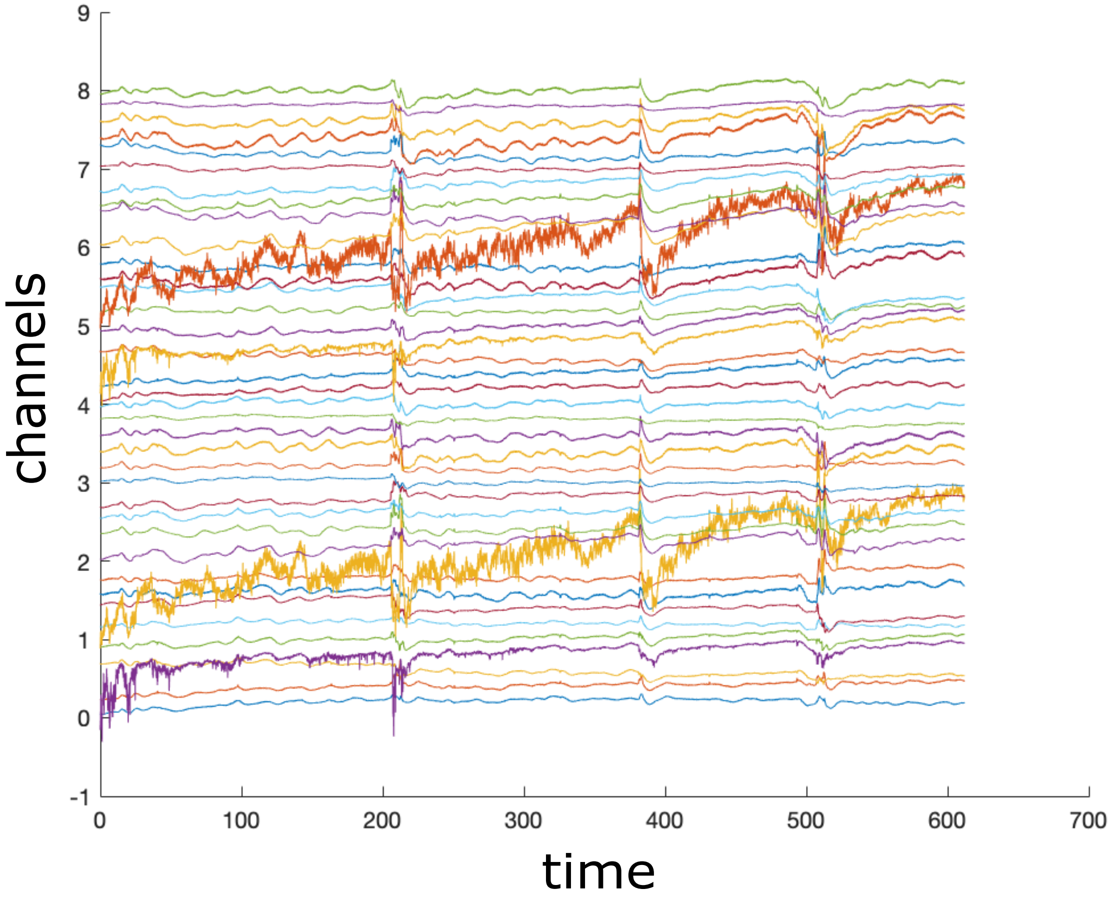

Correlation Plots

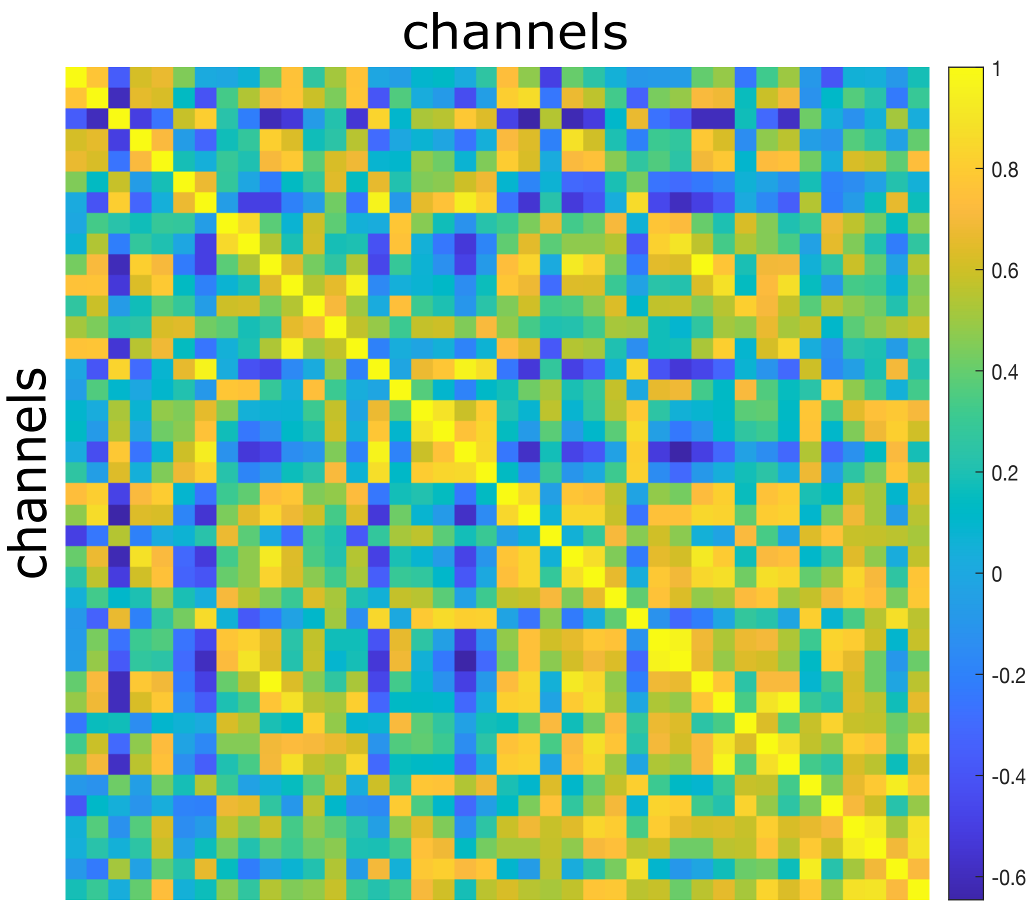

Remove Channels Without Cardiac

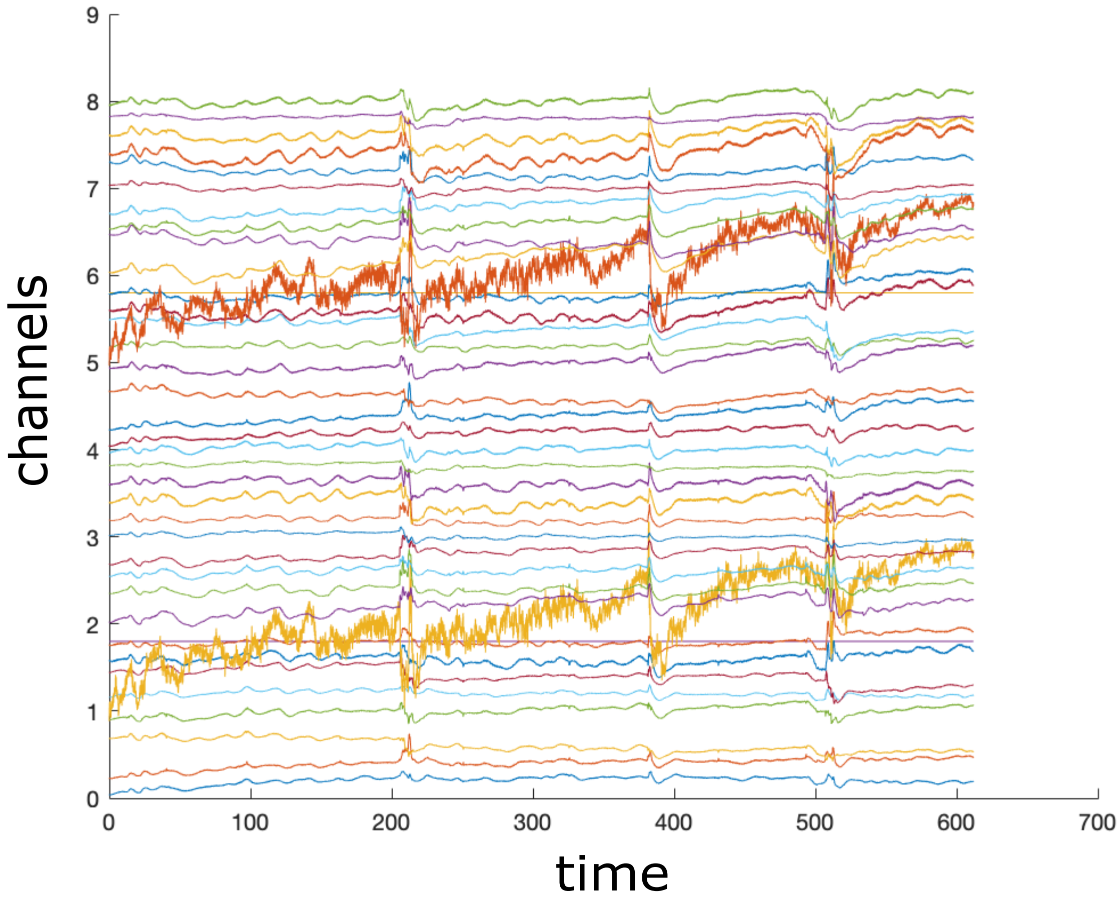

Correlation Plots

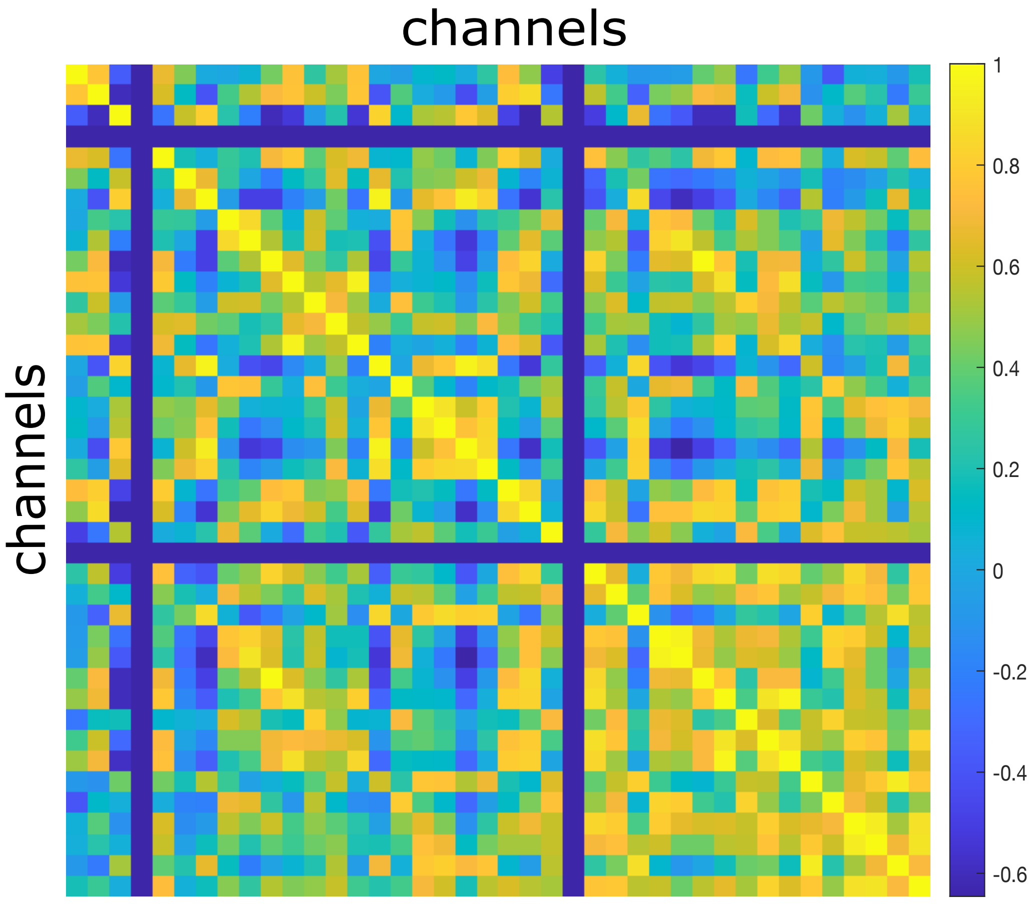

Segmentation

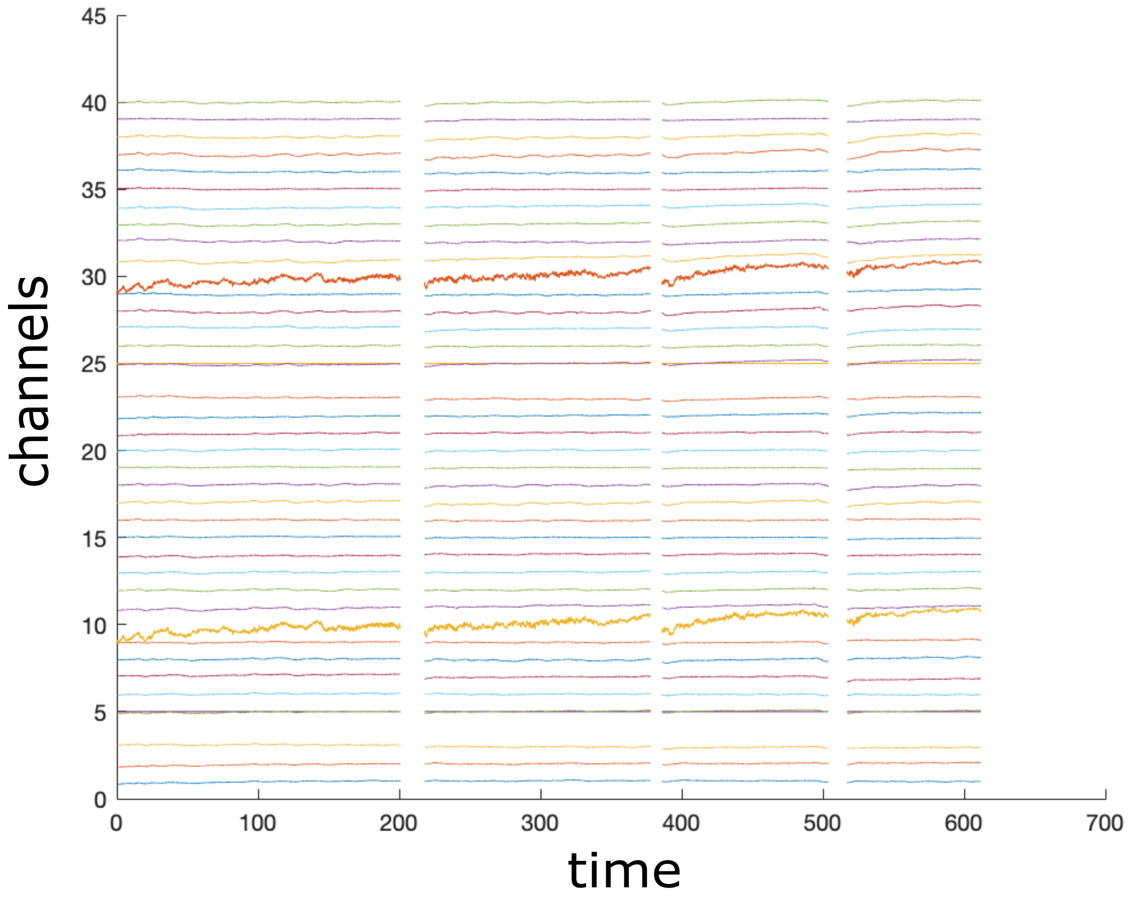

Correlation Plots

Segment 1

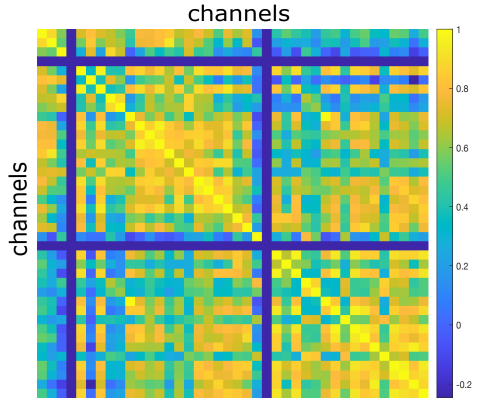

Segment 3

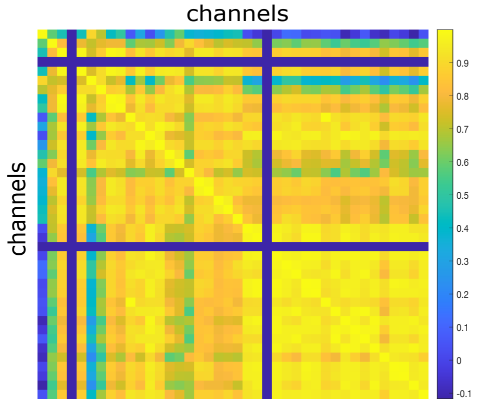

Segment 2

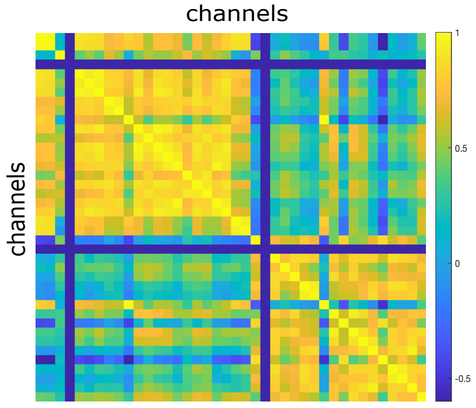

Segment 4

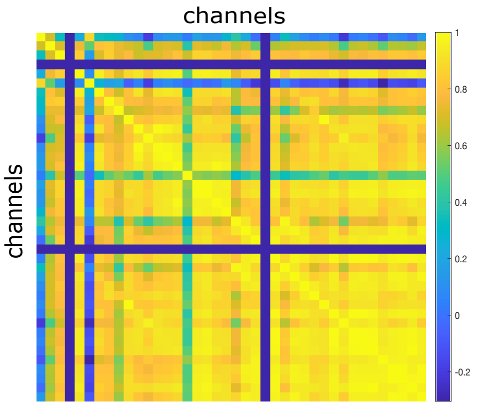

Wavelet Filter

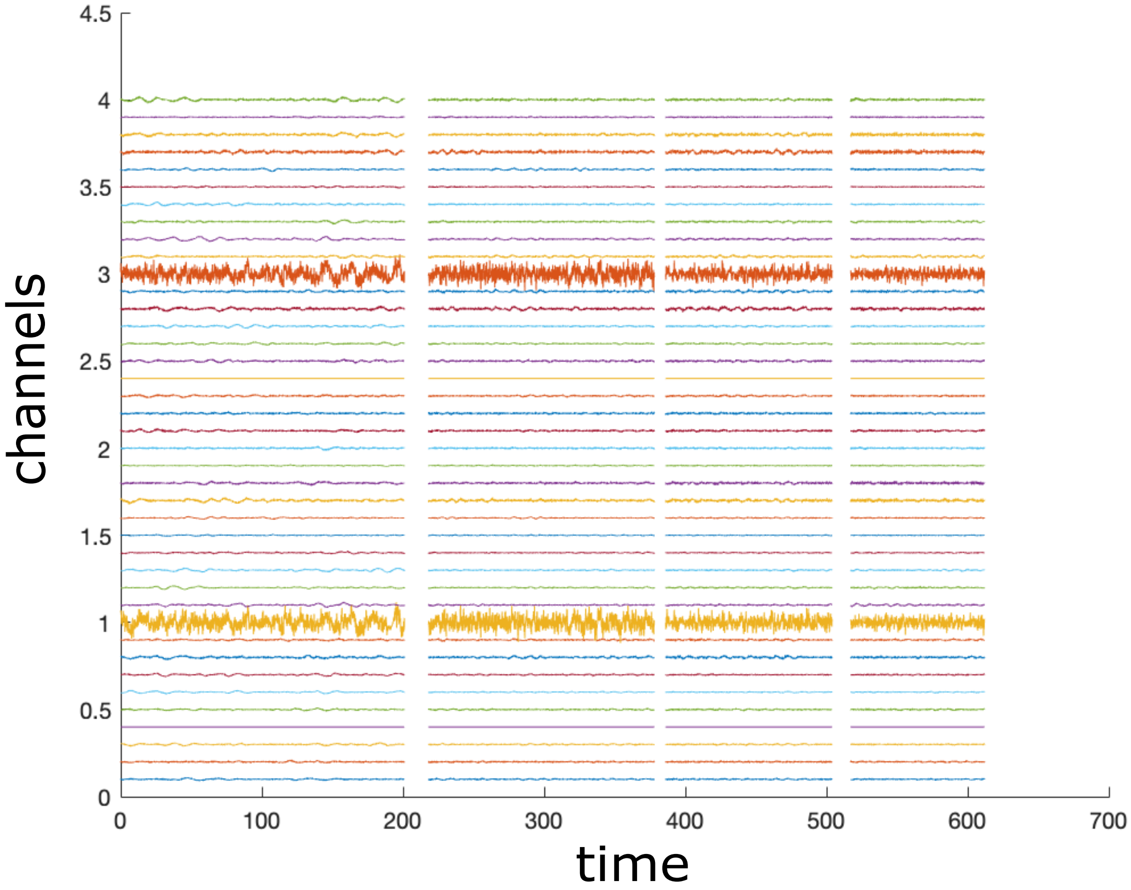

Correlation Plots

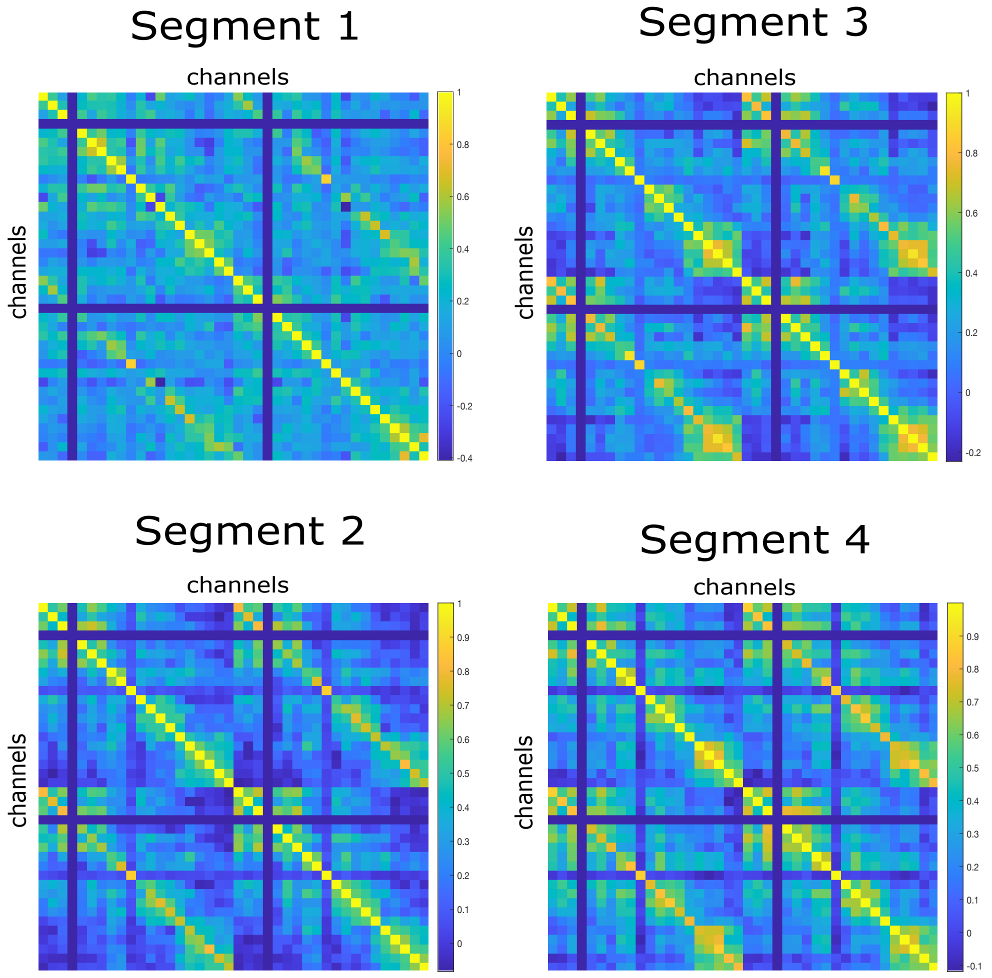

HbO and HbR

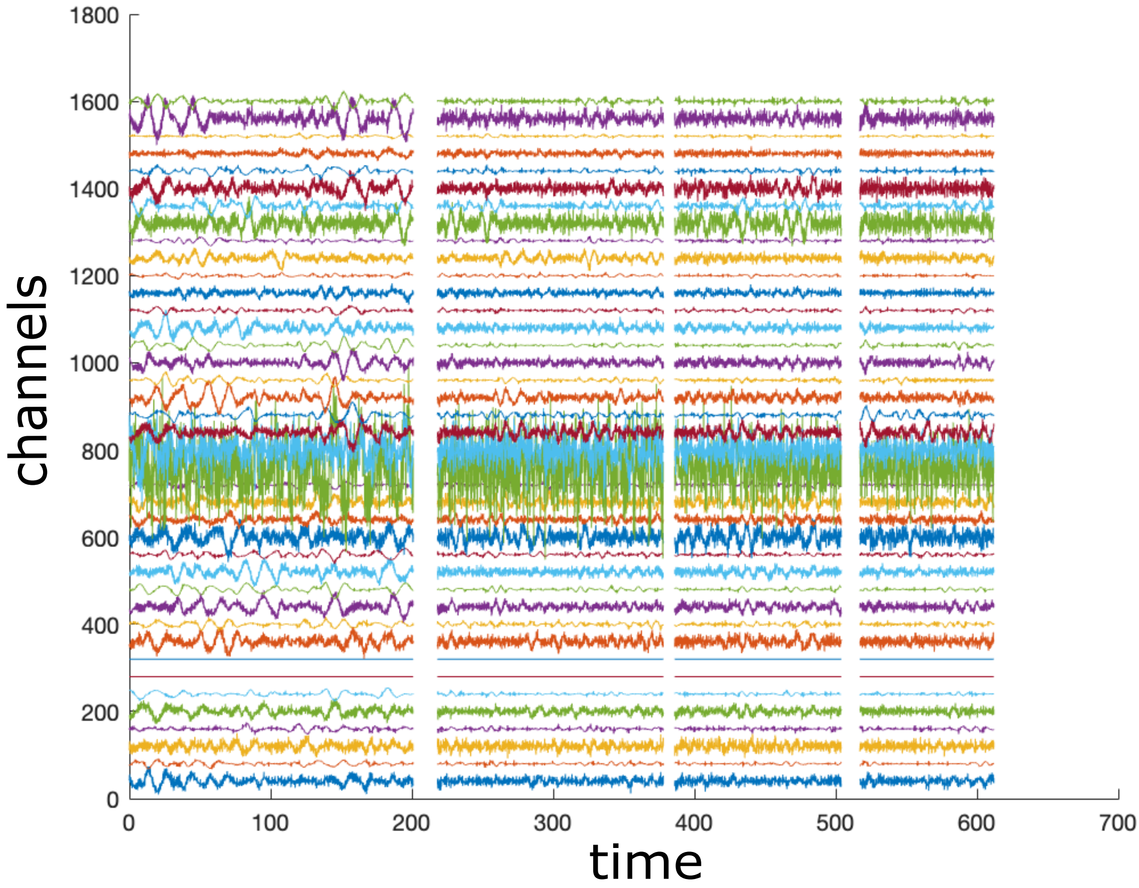

Correlation Plots

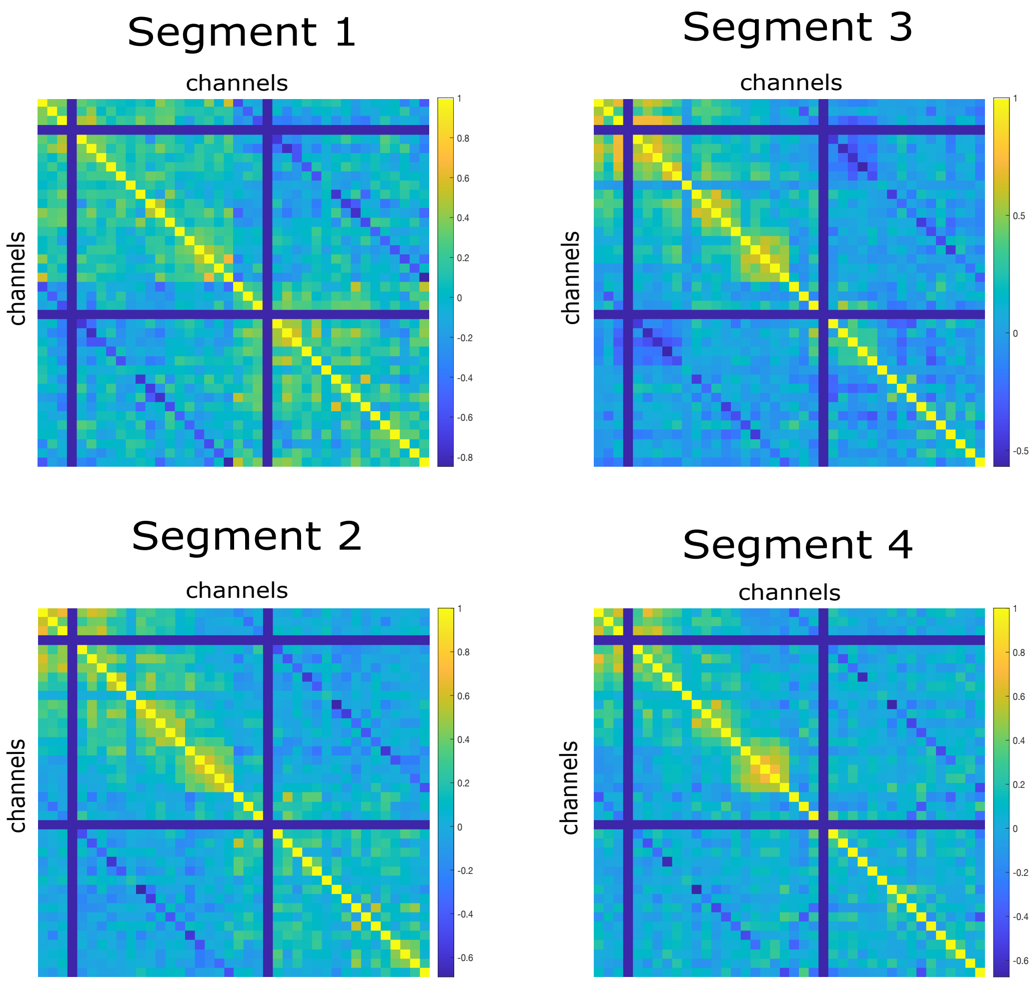

Bandpass Filter

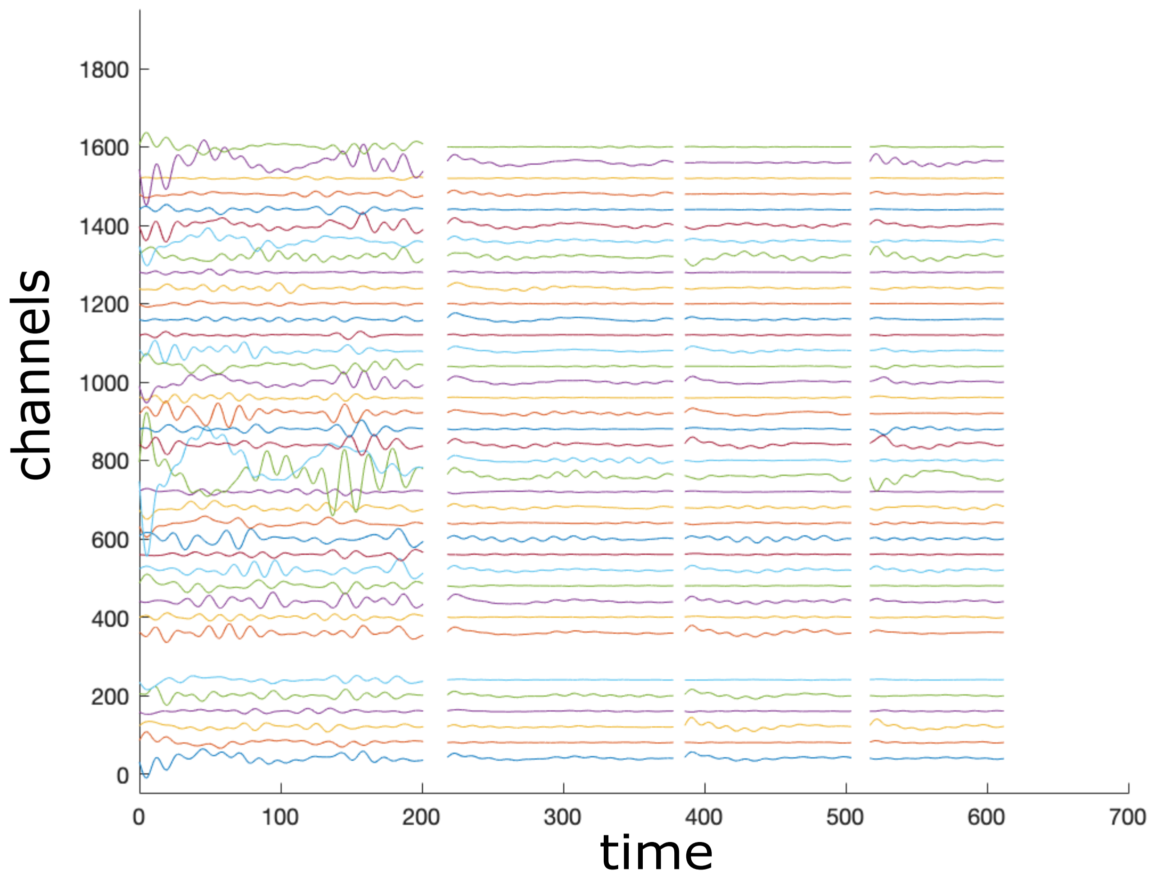

Correlation Plots

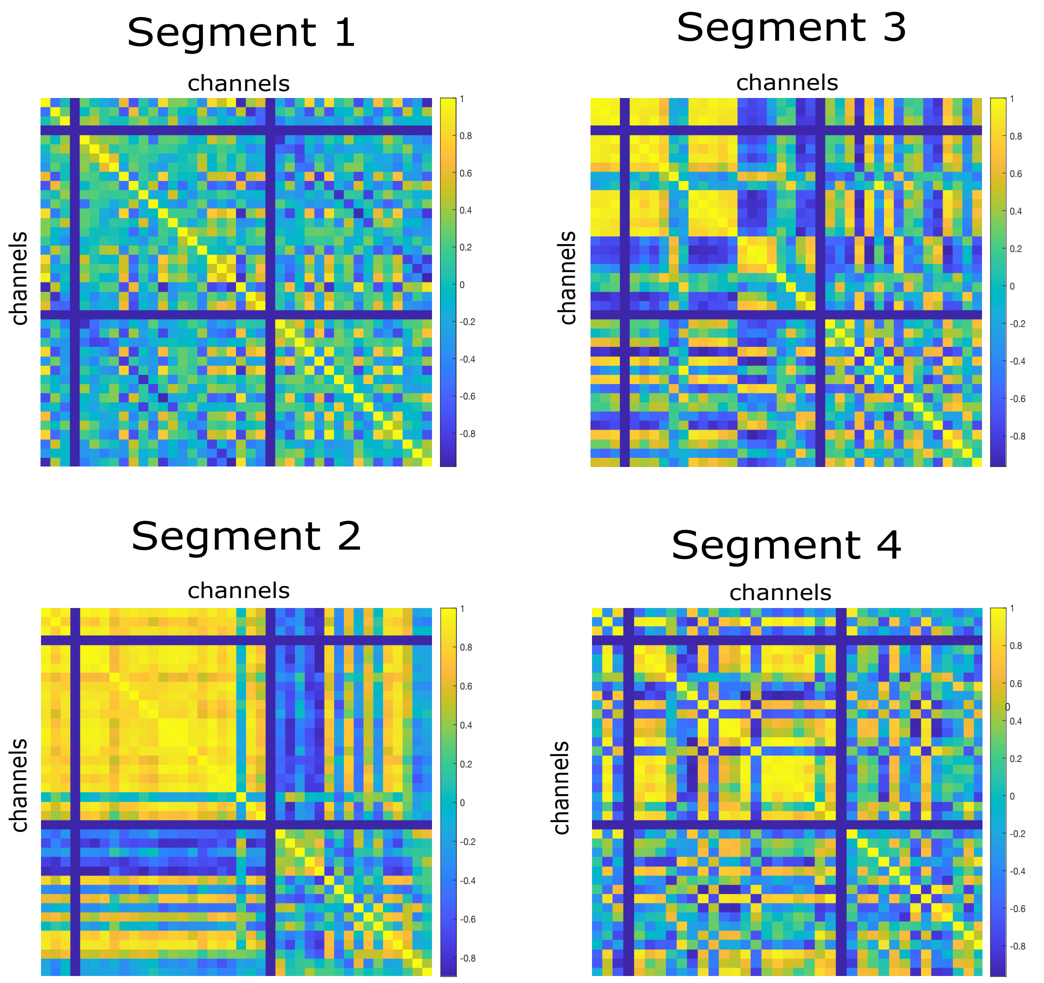

HbT

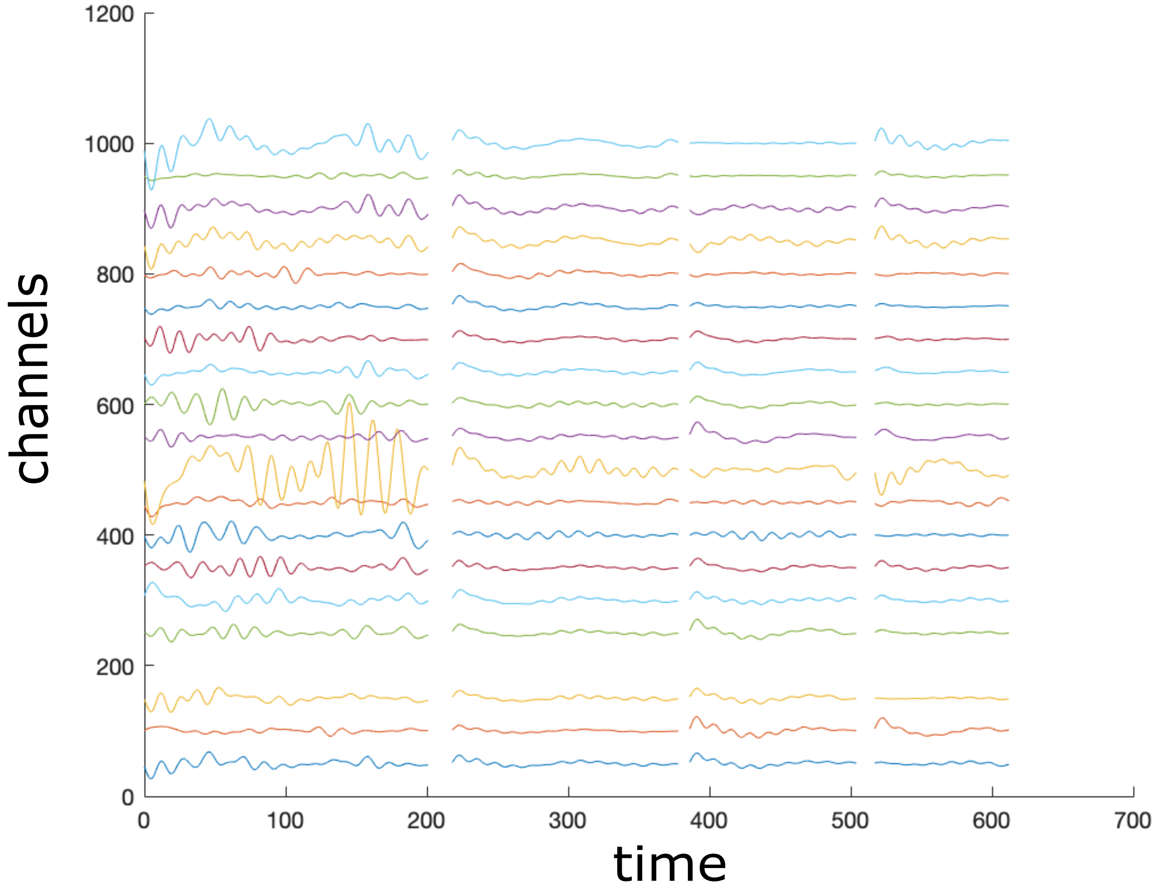

Correlation Plots

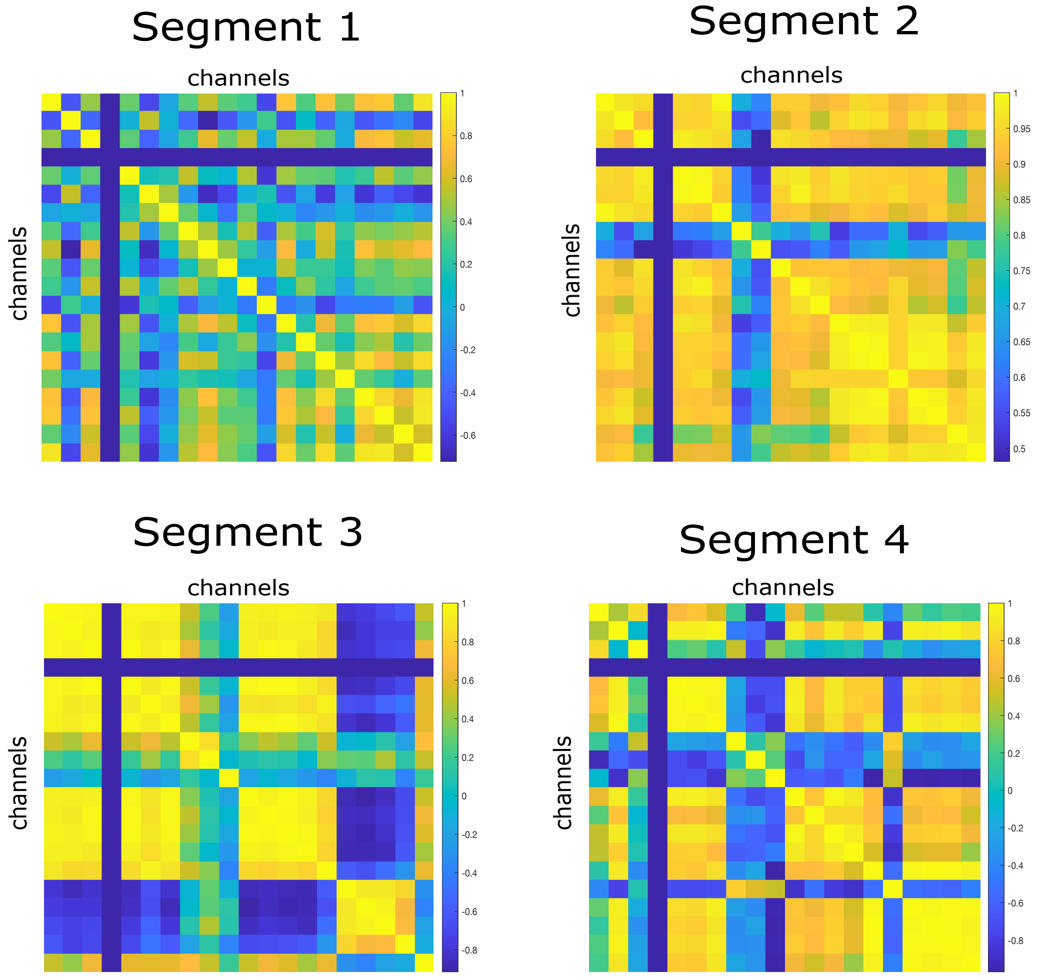

Merge Segments

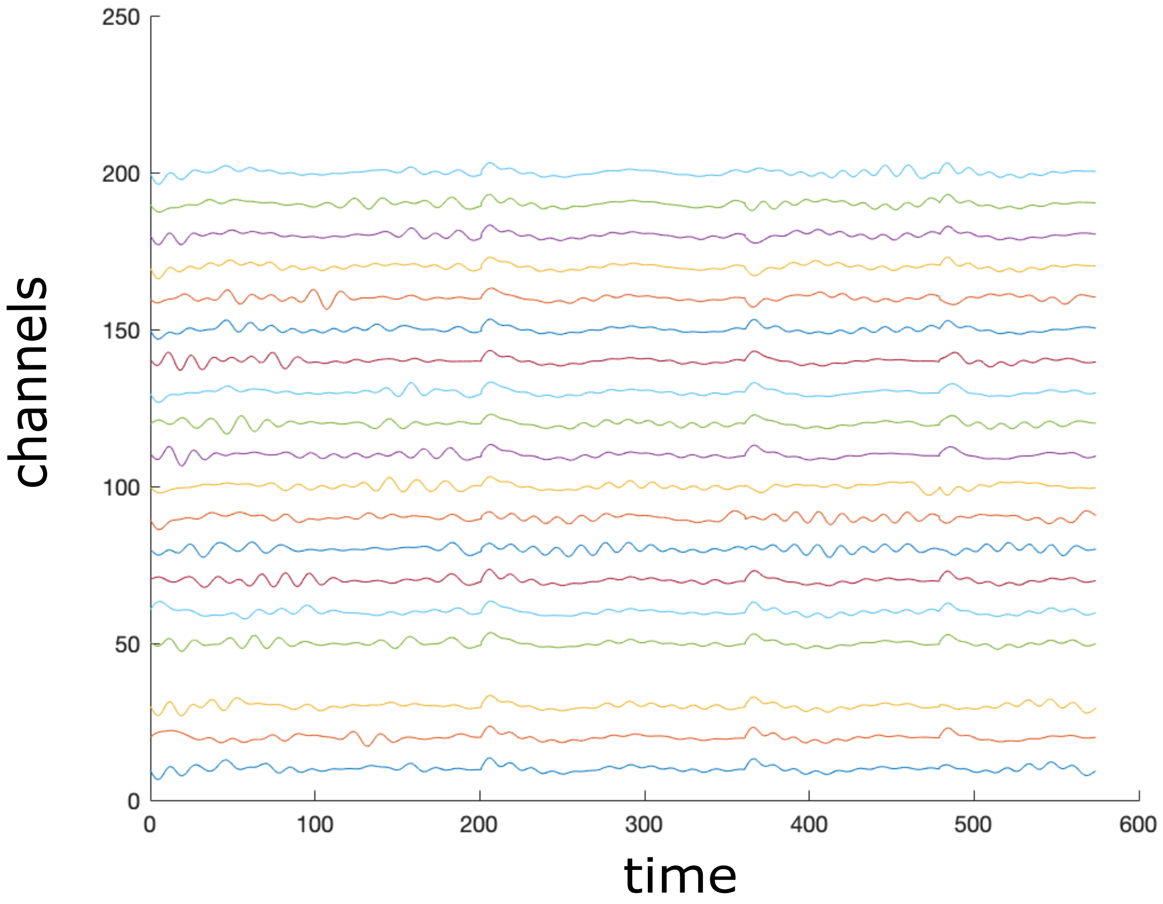

Correlation Plots

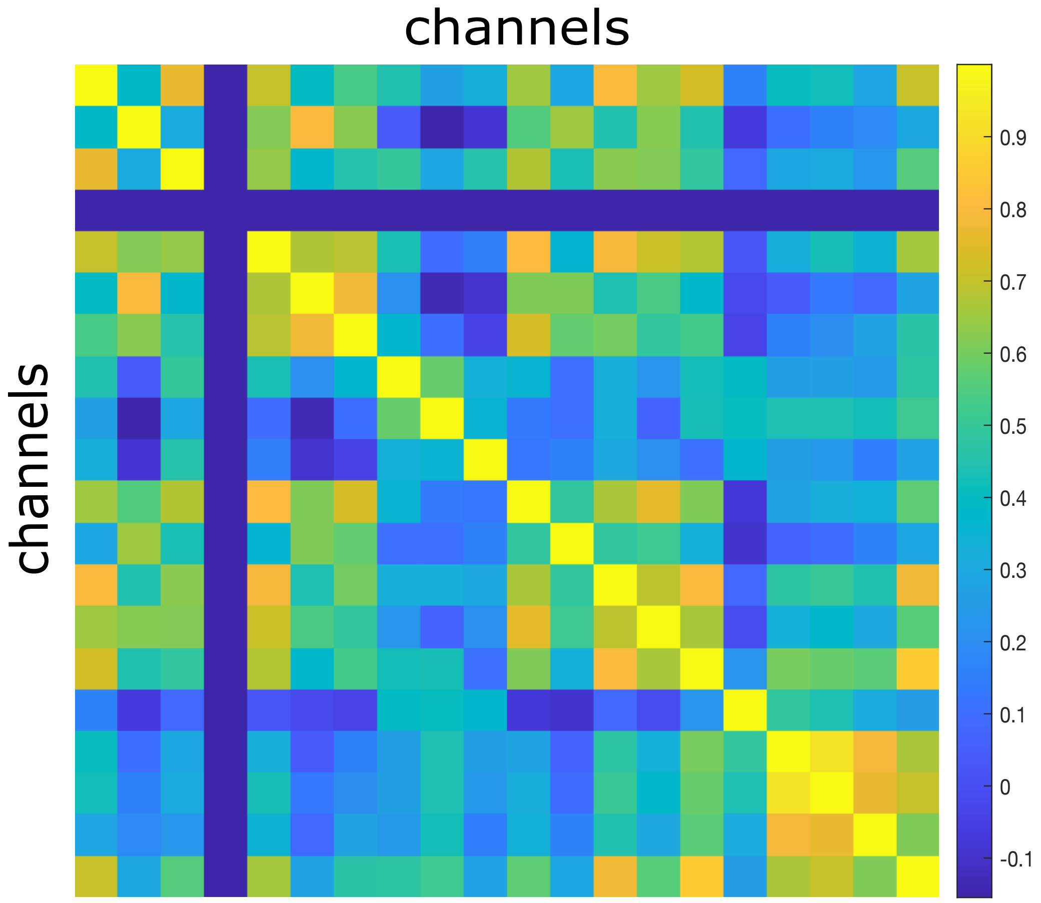

Resample

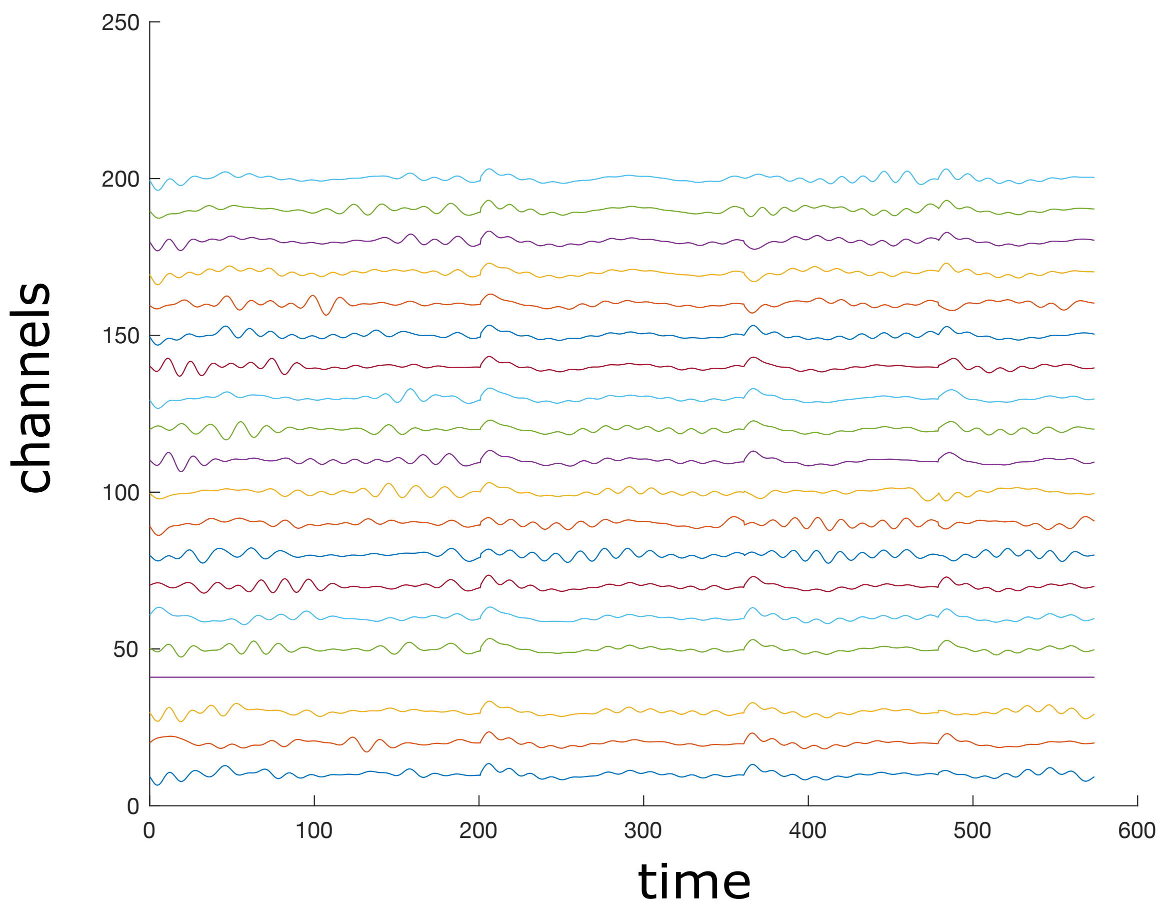

Correlation Plots

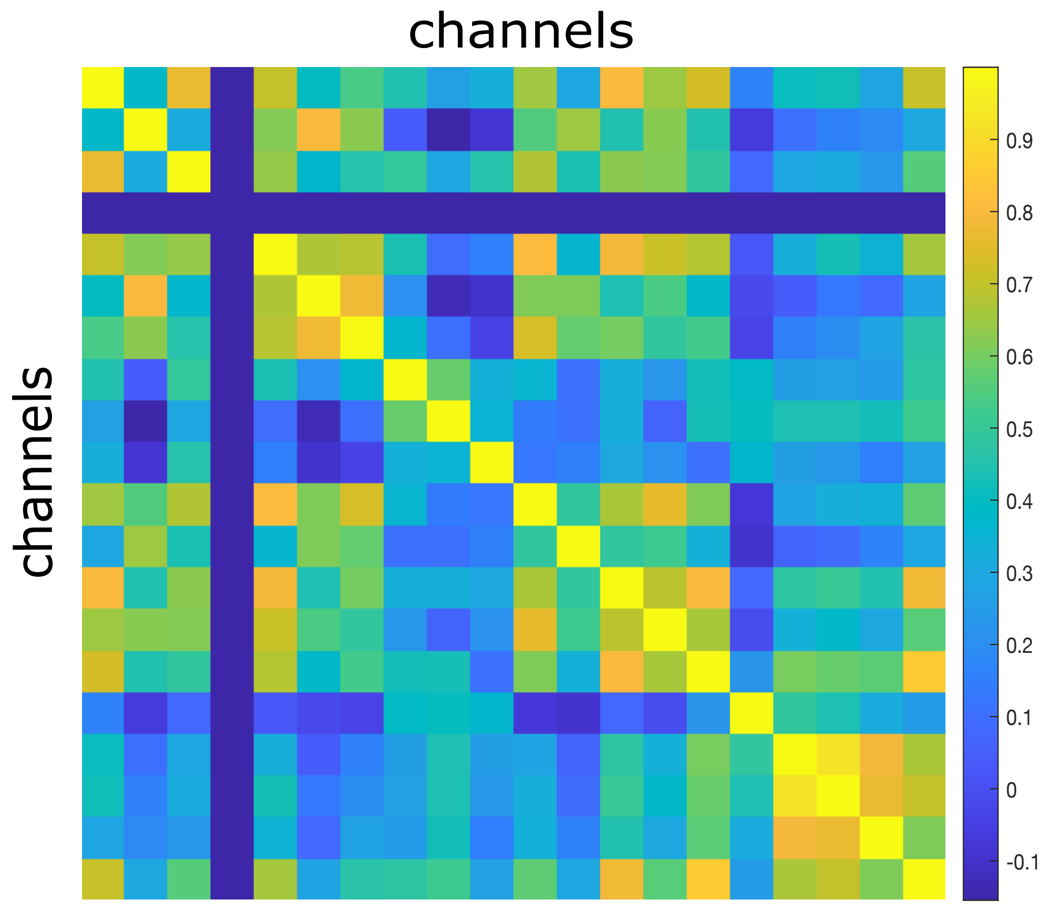

Supplement: Supplementary file 1 — Appendix 1. An overview of an example dataset at every processing stage. [file BRB3-14-e70180-s001.pdf]
