# Supplementary material for: Investigating Task‐Free Functional Connectivity Patterns in Newborns Using Functional Near‐Infrared Spectroscopy
Source: Brain Behav. 2024 Dec 17;14(12):e70180. doi: 10.1002/brb3.70180 (PMC11652786; doi:10.1002/brb3.70180)
Supplement: Supplementary file 2 — Figure S1. Group spatial map of channel exclusions (n = 41). Figure S2. Matrix showing group spontaneous functional connectivity (sFC) for HbT (n = 41). Figure S3. Matrix showing group spontaneous functional connectivity (sFC) for HbO (n = 41). Figure S4. Group t‐map showing spontaneous functional connectivity (sFC) spatial patterns for HbO (n = 41). Figure S5. Matrix showing group spontaneous functional connectivity (sFC) for HbR (n = 41). Figure S6. Group t‐map showing spontaneous functional connectivity (sFC) spatial patterns for HbR (n = 41). Figure S7. Matrix demonstrating gestational age–related patterns in spontaneous functional connectivity (sFC) (n = 41). Figure S8. Matrix demonstrating postnatal age–related patterns in spontaneous functional connectivity (sFC) (n = 41). [file BRB3-14-e70180-s002.docx]

**Supplementary Materials**

**
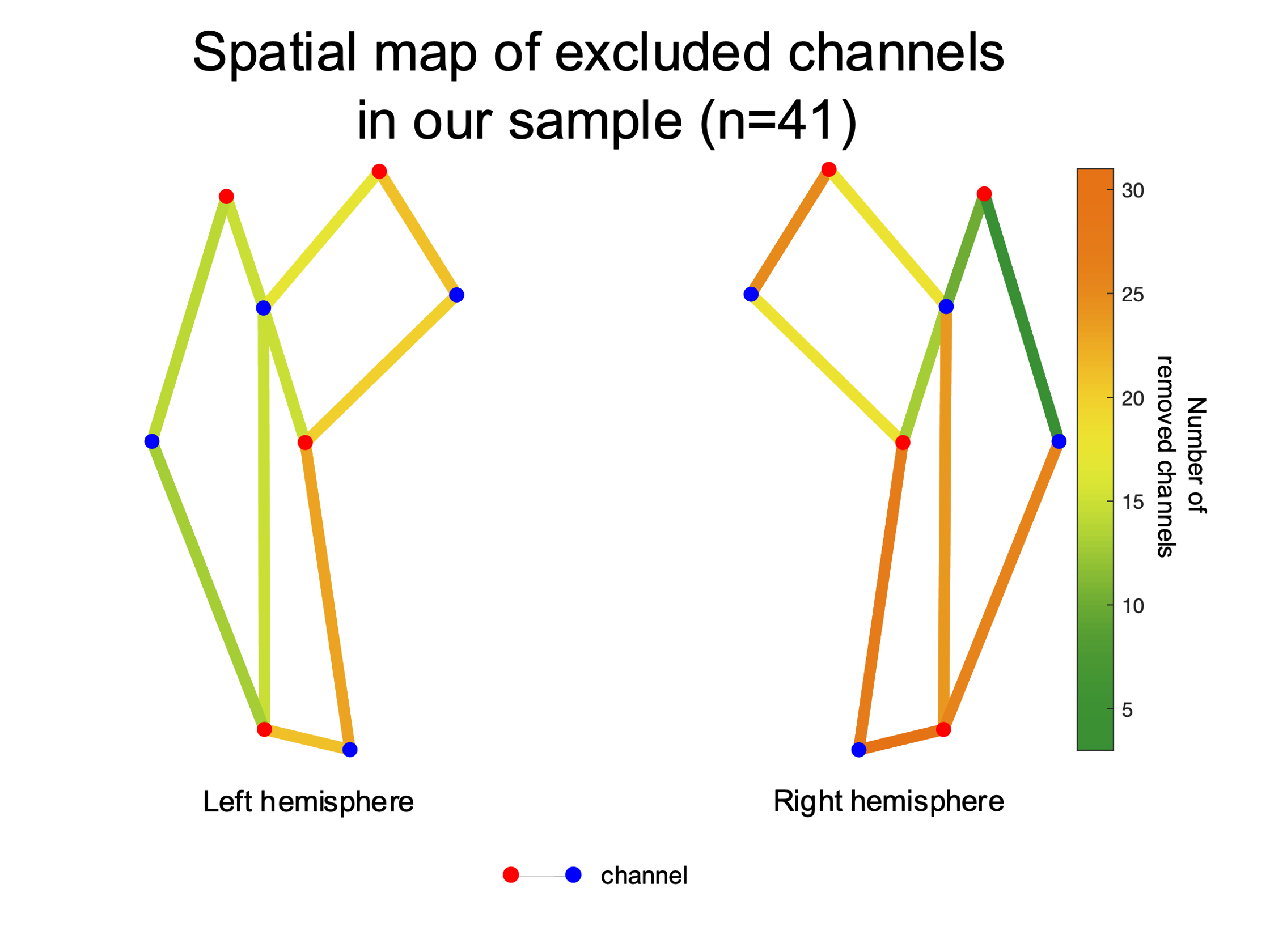
**

**Figure S1. Group spatial map of channel exclusions (n=41).** The color of the lines depicts the number of infants in which that channel was excluded. Channels depicted in green were preserved in most infants while channels depicted in orange were excluded in most infants.

**Figure S2. Matrix showing group spontaneous functional connectivity (sFC) for HbT (n=41).** False discovery rate (FDR) was used to correct for multiple comparisons. Channel-pairs that exhibited significant connectivity after FDR correction are highlighted using ** while channel-pairs with significant connectivity before FDR correction are highlighted using *. The color of matrix cells represents the t-value calculated for that channel-pair’s connectivity. The diagonal is not informative.

**Figure S3. Matrix showing group spontaneous functional connectivity (sFC) for HbO (n=41).** False discovery rate (FDR) was used to correct for multiple comparisons. Channel-pairs that exhibited significant connectivity after FDR correction are highlighted using ** while channel-pairs with significant connectivity before FDR correction are highlighted using *. The color of matrix cells represents the t-value calculated for that channel-pair’s connectivity. The diagonal is not informative.

**
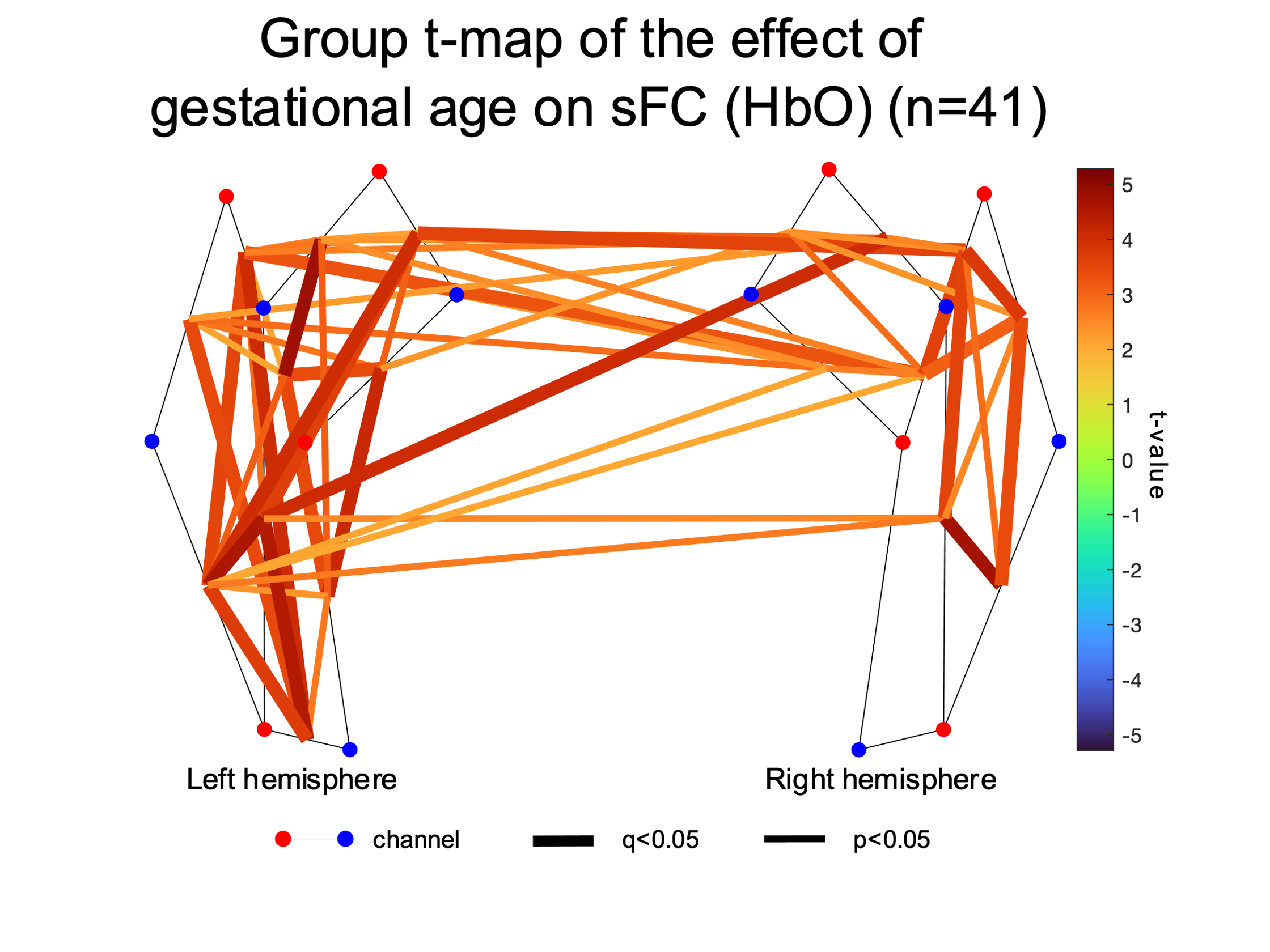
**

**Figure S4. Group t-map showing spontaneous functional connectivity (sFC) spatial patterns for HbO (n=41).** Channel-pairs displaying a significant positive or negative sFC are depicted in red and blue lines, respectively. The false discovery rate (FDR) was used to correct for multiple comparisons. Channel-pairs that exhibited significant connectivity after FDR correction are drawn as thick lines while channel-pairs with significant connectivity before FDR correction are denoted with thin lines. The color of the lines represents the t-value calculated for that channel-pair’s connectivity. Channel-pairs where one or both channels were missing (due to subpar quality) were not included in the analysis. Channel-pairs that had fewer than 10 datapoints and those that were not significant have been omitted to increase clarity.

**Figure S5. Matrix showing group spontaneous functional connectivity (sFC) for HbR (n=41).** False discovery rate (FDR) was used to correct for multiple comparisons. Channel-pairs that exhibited significant connectivity after FDR correction are highlighted using ** while channel-pairs with significant connectivity before FDR correction are highlighted using *. The color of matrix cells represents the t-value calculated for that channel-pair’s connectivity. The diagonal is not informative.

**
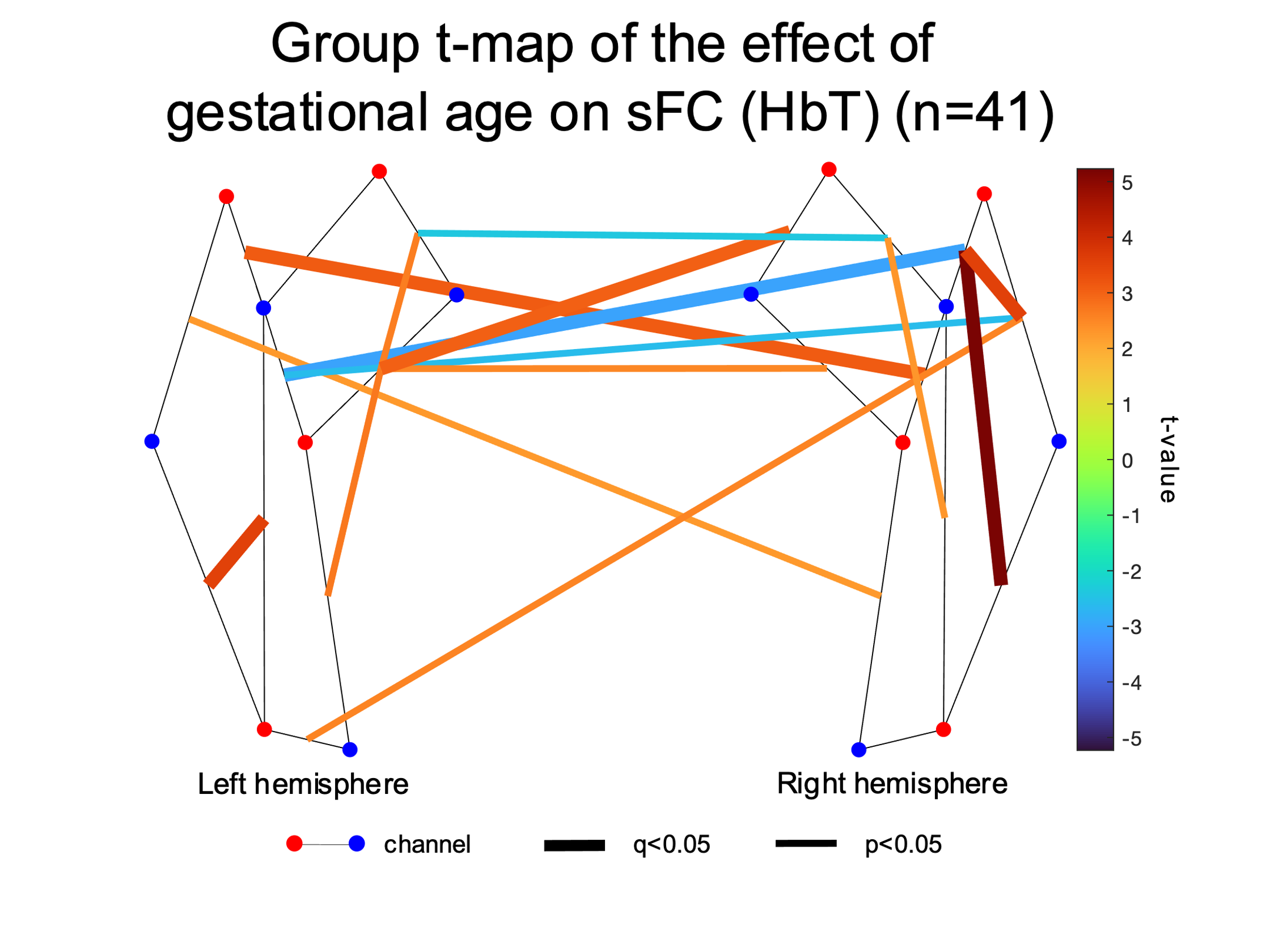
**

**Figure S6. Group t-map showing spontaneous functional connectivity (sFC) spatial patterns for HbR (n=41).** Channel-pairs displaying a significant positive or negative sFC are depicted in red and blue lines, respectively. The false discovery rate (FDR) was used to correct for multiple comparisons. Channel-pairs that exhibited significant connectivity after FDR correction are drawn as thick lines while channel-pairs with significant connectivity before FDR correction are denoted with thin lines. The color of the lines represents the t-value calculated for that channel-pair’s connectivity. Channel-pairs where one or both channels were missing (due to subpar quality) were not included in the analysis. Channel-pairs that had fewer than 10 datapoints and those that were not significant have been omitted to increase clarity.

**Figure S7. Matrix demonstrating gestational-age related patterns in spontaneous functional connectivity (sFC) (n=41).** False discovery rate (FDR) was used to correct for multiple comparisons. Channel-pairs whose connectivity exhibited a significant relationship with gestational age after FDR correction are highlighted using ** while channel-pairs showing a significant relationship before FDR correction are highlighted using *. The color of matrix cells represents the t-value calculated for that channel-pair. The diagonal is not informative.

**Figure S8. Matrix demonstrating postnatal-age related patterns in spontaneous functional connectivity (sFC) (n=41).** False discovery rate (FDR) was used to correct for multiple comparisons. Channel-pairs whose connectivity exhibited a significant relationship with postnatal age after FDR correction are highlighted using ** while channel-pairs showing a significant relationship before FDR correction are highlighted using *. The color of matrix cells represents the t-value calculated for that channel-pair. The diagonal is not informative.
